# Calcium ions trigger the exposure of phosphatidylserine on the surface of necrotic cells

**DOI:** 10.1371/journal.pgen.1009066

**Published:** 2021-02-11

**Authors:** Yoshitaka Furuta, Omar Pena-Ramos, Zao Li, Lucia Chiao, Zheng Zhou

**Affiliations:** 1 Verna and Marrs McLean Department of Biochemistry and Molecular Biology, Baylor College of Medicine, Houston, Texas, United States of America; 2 School of Pharmacy, Kanazawa University, Kakuma-machi, Kanazawa, Ishikawa, Japan; University of California San Francisco, UNITED STATES

## Abstract

Intracellular Ca^2+^ level is under strict regulation through calcium channels and storage pools including the endoplasmic reticulum (ER). Mutations in certain ion channel subunits, which cause mis-regulated Ca^2+^ influx, induce the excitotoxic necrosis of neurons. In the nematode *Caenorhabditis elegans*, dominant mutations in the DEG/ENaC sodium channel subunit MEC-4 induce six mechanosensory (touch) neurons to undergo excitotoxic necrosis. These necrotic neurons are subsequently engulfed and digested by neighboring hypodermal cells. We previously reported that necrotic touch neurons actively expose phosphatidylserine (PS), an “eat-me” signal, to attract engulfing cells. However, the upstream signal that triggers PS externalization remained elusive. Here we report that a robust and transient increase of cytoplasmic Ca^2+^ level occurs prior to the exposure of PS on necrotic touch neurons. Inhibiting the release of Ca^2+^ from the ER, either pharmacologically or genetically, specifically impairs PS exposure on necrotic but not apoptotic cells. On the contrary, inhibiting the reuptake of cytoplasmic Ca^2+^ into the ER induces ectopic necrosis and PS exposure. Remarkably, PS exposure occurs independently of other necrosis events. Furthermore, unlike in mutants of DEG/ENaC channels, in dominant mutants of *deg-3* and *trp-4*, which encode Ca^2+^ channels, PS exposure on necrotic neurons does not rely on the ER Ca^2+^ pool. Our findings indicate that high levels of cytoplasmic Ca^2+^ are necessary and sufficient for PS exposure. They further reveal two Ca^2+^-dependent, necrosis-specific pathways that promote PS exposure, a “two-step” pathway initiated by a modest influx of Ca^2+^ and further boosted by the release of Ca^2+^ from the ER, and another, ER-independent, pathway. Moreover, we found that ANOH-1, the worm homolog of mammalian phospholipid scramblase TMEM16F, is necessary for efficient PS exposure in thapsgargin-treated worms and *trp-4* mutants, like in *mec-4* mutants. We propose that both the ER-mediated and ER-independent Ca^2+^ pathways promote PS externalization through activating ANOH-1.

## Author summary

Necrosis is a type of cell death that exhibits distinct morphological features such as cell and organelle swelling. Necrotic cells expose phosphatidylserine (PS)–a type of phospholipid—on their outer surfaces. Receptor molecules on phagocytes detect PS on necrotic cells and subsequently initiate the engulfment process. As necrosis is associated with stroke, cancer, neurodegenerative diseases, and heart diseases, studying necrotic cell clearance has important medical relevance. In the model organism the nematode *C*. *elegans*, we previously identified membrane proteins that promote the exposure of PS on necrotic cell surfaces by studying neurons that are induced to undergo necrosis by dominant mutations in ion channels. Here, in *C*. *elegans*, we have discovered that the necrotic insults trigger an increase of the cytoplasmic calcium ion (Ca^2+^), which in turn promotes PS externalization on necrotic cell surfaces. Furthermore, we have identified two different mechanisms that increase cytoplasmic Ca^2+^ levels, one dependent on the Ca^2+^ contribution from the endoplasmic reticulum (ER), the other independent of the ER. The Ca^2+^ signal targets ANOH-1, a worm homolog of mammalian proteins capable of externalizing PS, for promoting PS exposure on necrotic cells. Our findings reveal novel upstream regulatory mechanisms that promote necrotic cell clearance in animals.

## Introduction

Cells undergoing necrosis—a type of cell death morphologically distinct from apoptosis—display cell and organelle swelling, excessive intracellular membranes, and the eventual rupture of the intracellular and plasma membranes [1,2]. Necrosis is frequently observed during cell injury, and is closely associated with stroke, heart diseases, inflammatory diseases, diabetes, cancer, and neurodegeneration [3–8]. Although necrosis has historically been considered an uncontrolled cell death event, recent discoveries from multiple organisms demonstrated that cells possess genetic programs that trigger necrosis in response to extracellular stimuli [9–12]. In the nematode *Caenorhabditis elegans*, dominant (*d*) mutations in certain ion channel subunits of the DEG/ENaC (degenerin/epithelial sodium channel) superfamily, in the nicotinic acetylcholine receptor, in trimeric GTPases, and in a few other proteins induce neurons of specific identities to undergo a type of necrosis that resembles the excitotoxic necrosis of mammalian neurons [10]. One such gene is *mec-4*, which encodes a core subunit of a multimeric, mechanically gated sodium channel that belongs to the DEG/ENaC family and functions in the mechanosensory (touch) neurons for sensing gentle touch along the worm body [13]. Dominant mutations in *mec-4* trigger the necrosis of all six touch neurons (AVM, PVM, ALM(L/R) and PLM(L/R)) [13,14]. Unlike apoptosis, the necrosis triggered by the *mec-4(d)* mutations does not require the function of CED-3, the *C*. *elegans* caspase [15]. Furthermore, many of the cellular events occurring during necrosis are different from those occurring during apoptosis [1,2].

Cells undergoing necrosis, like those undergoing apoptosis, are recognized, engulfed, and degraded by neighboring engulfing cells [16,17]. Apoptotic cells are known to present phosphatidylserine (PS), a membrane phospholipid, on their outer surfaces to attract the phagocytic receptors on engulfing cells [18]. PS is thus referred to as an “eat me” signal recognized by engulfing cells and triggering subsequent engulfment [18]. We previously discovered that the outer surfaces of necrotic touch neurons in *mec-4(d)* mutant worms expose PS, like apoptotic cells [19,20]. As a consequence, PS interacts with the phagocytic receptor CED-1 residing on the surfaces of neighboring engulfing cells, allowing necrotic cells to be recognized by engulfing cells [19]. We found that PS was actively exposed on the surface of necrotic neurons while the plasma membrane remained intact [19]. In addition, we identified two proteins that act in parallel to promote the externalization of PS from the inner leaflet of the plasma membrane to the outer leaflet during necrosis [19]. One of these proteins is CED-7, the *C*. *elegans* homologs of the mammalian ABCA transporter [19,21]. Mammalian ABCA possesses the PS-externalization activity [22], and CED-7 is also essential for promoting PS exposure on the outer surfaces of apoptotic cells [20,23]. The other protein is ANOH-1, the *C*. *elegans* homolog of mammalian phospholipid scramblase TMEM16F, which catalyzes the random, bi-directional “flip-flop” of phospholipids across the membrane bilayer [19,24]. Whereas CED-7 is expressed broadly and its function needed in both apoptotic and necrotic cells for efficient PS exposure, ANOH-1 is specifically expressed in neurons and acts specifically in necrotic neurons, but not apoptotic cells, to facilitate PS exposure [19–21]. Mammalian TMEM16F was reported to promote PS exposure on the surfaces of platelets during the blood clotting process [24–26]. To our knowledge, *C*. *elegans* ANOH-1 is the first phospholipid scramblase reported to promote PS exposure on necrotic cells.

The proteins responsible for PS externalization on *C*. *elegans* touch neurons during necrosis are presumably activated by upstream signal(s). However, the identity of such upstream signal(s) remains unknown. In comparison, during apoptosis, caspases are known to be the critical upstream molecules that trigger the exposure of PS and other cellular events. In living cells, PS is almost exclusively localized to the inner leaflet of the plasma membrane [18]. In mammalian cells undergoing apoptosis, caspase 3 cleaves and inactivates the aminophospholipid translocase ATP11C, which selectively flips PS from the outer to the inner leaflet in living cells [27]. The mammalian caspase 3 and *C*. *elegans* CED-3 cleave and activate Xk-related protein 8 (Xk8), also a phospholipid scramblase, and Xk8’s worm homolog CED-8, respectively [28,29].

Caspase activity is not involved in excitotoxic necrosis, including the necrosis of the touch neurons triggered by *mec-4(d)* mutations [15,30,31]. Rather, intracellular calcium ions were considered important signaling molecules that induce the necrosis of multiple types of cells [32–34], including the *C*. *elegans* touch neurons [35]. The *mec-4(d)* mutations alter the conformation of the mechanosensory Na^+^ channel (of which MEC-4 is a subunit) and make it permeable to extracellular Ca^2+^ [36]. Remarkably, *mec-4(d)* induced necrosis is partially suppressed by loss-of-function (*lf*) mutations in *crt-1 –*which encodes calreticulin, the ER residential Ca^2+^ chaperone that facilitates the accumulation of Ca^2+^ in the ER—and by inactivating genes encoding the ER Ca^2+^-release channels [35]. Additionally, *mec-4(d)*-induced necrosis is suppressed by chemical treatment that impairs the release of Ca^2+^ from the ER to the cytoplasm [35]. Based on the knowledge that (1) the cytoplasmic Ca^2+^ level is generally much lower than that in the extracellular space or intracellular Ca^2+^ pools [37], (2) the ER is one of the primary storage pools for intracellular Ca^2+^ [37], and (3) the Ca^2+^ release channels in the ER are activated by the increase of cytoplasmic Ca^2+^ [38], Xu et al [35] proposed that the small amount of extracellular Ca^2+^ that entered the touch neurons through the MEC-4(d)-containing Na^+^ channel further induced a robust Ca^2+^ release from the ER to the cytoplasm. Moreover, they propose that necrosis is induced once the level of cytoplasmic Ca^2+^ reaches a certain threshold level [35]. Closely related to this discovery and to our investigation of PS exposure on necrotic cells, the scramblase activity of mammalian TMEM16F is known to be Ca^2+^-dependent [24].

We set out to investigate whether cytoplasmic Ca^2+^ triggers PS exposure on necrotic cells and whether the ER, as an intracellular Ca^2+^ pool, takes part in the externalization of PS. Using a fluorescently tagged-MFG-E8, a secreted reporter that detects PS on the outer surfaces of cells [19,20], we quantitatively monitored PS exposure on the cell surface. Previously, the level of Ca^2+^ in touch neurons has not been monitored over the course of necrosis. To study the relationship between cytoplasmic Ca^2+^ and PS exposure, we developed a touch neuron-specific Ca^2+^ indicator. By monitoring the Ca^2+^ indicator and the PS reporter, we observed a close relationship between a surge of cytoplasmic Ca^2+^ and the subsequent exposure of PS on the cell surface of necrotic cells. We further determined that the exposure of PS was regulated by Ca^2+^. Moreover, we found that the release of Ca^2+^ from the ER was essential for the exposure of PS in mutants of the DEC/ENaC sodium channel subunits, yet not necessary for that in mutants of two different Ca^2+^ channels. In addition, PS exposure is independent of cell swelling, another necrosis event. Lastly, the efficient Ca^2+^-triggered PS exposure depends on the function of ANOH-1. Our findings reveal necrotic cell-specific, Ca^2+^-mediated regulatory mechanisms that act to trigger the exposure of the “eat me” signal and the clearance of necrotic cells.

## Results

### Multiple kinds of necrotic neurons expose phosphatidylserine (PS) on their surfaces

**Fig 1:**
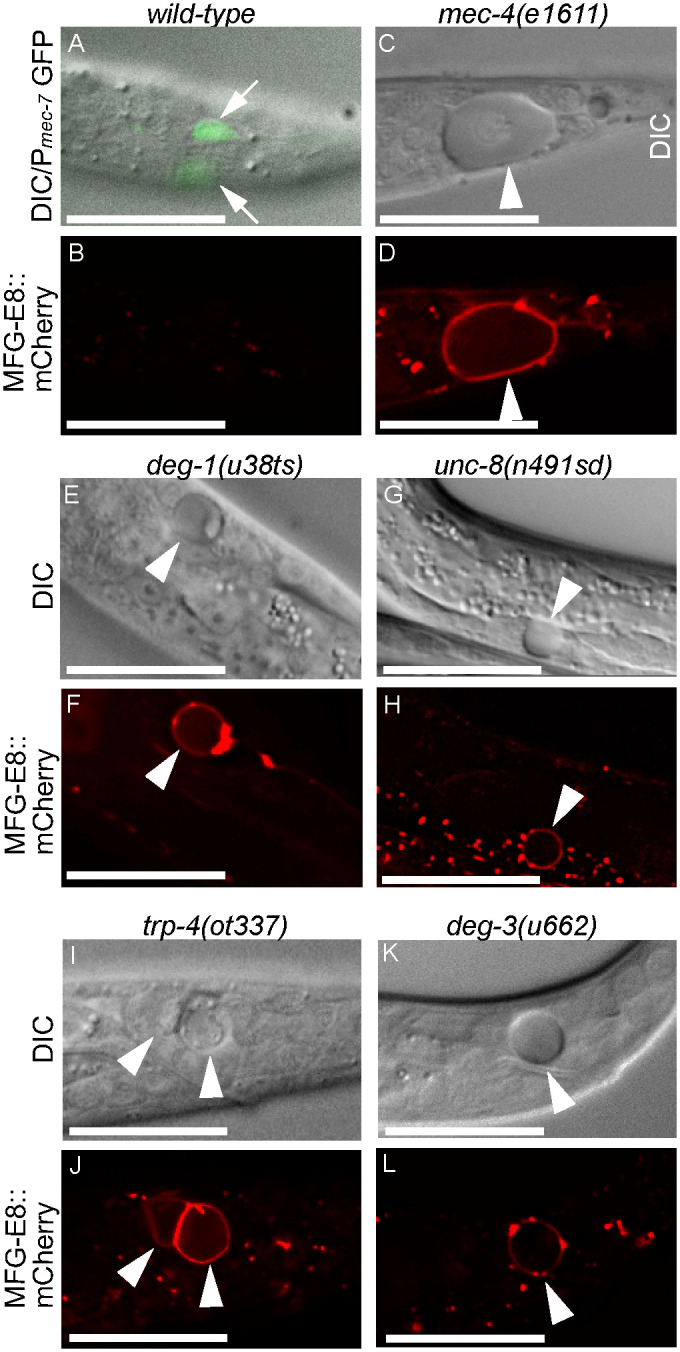
PS exposure is a common phenomenon observed on various necrotic neurons. DIC (A, C, E, G, I, K) and epifluorescence (B, D, F, H, J, L) images of two live touch neurons (white arrows) and six necrotic neurons (white arrowheads) in the L1 larvae of wild-type and different mutant strains expressing *P*_*dyn-1*_*mfg-e8*::*mCherry*, including the PLM touch neuron in the tail (A-D), the AVG interneuron in the head (E-F), a ventral motor neuron (G-H), the DVA and DVC neurons in the tail (I-J), and a putative PVD neuron in the tail (K-L). In (A), P_*mec-7*_GFP labels two PLM neurons (white arrows). In (E-F), AVG interneurons in the head are observed at an incubation temperature of 25°C, as *deg-1(u38ts)* is a temperature sensitive mutant. The bright puncta seen in (B, D, F, H, J, L) are non-specific aggregates of MFG-E8::mCherry. Scale bars are 15μm.

The six *C*. *elegans* touch neurons, when undergoing necrosis in the *mec-4(e1611d)* mutant strain, expose PS on their surfaces [19]. This PS signal is detected by an mCherry-tagged MFG-E8, a PS-binding protein that is expressed in neighboring hypodermal cells and subsequently secreted to the extracellular space (Fig 1C and 1D) [19]. Conversely, MFG-E8::mCherry does not detect PS on the surfaces of live touch neurons (marked by P_*mec-7*_GFP) in wild-type larvae (Fig 1A and 1B) [19]. To determine whether PS exposure is a common feature for necrotic neurons of different kinds and induced to die by different insults, we examined a number of necrotic neurons in *deg-1(u38)*, *unc-8(n491sd)*, *trp-4(ot337)*, and *deg-3(u662)* dominant mutant L1 larvae. *deg-1* encodes a DEG/ENaC family sodium channel subunit involved in chemotaxis [39]. *unc-8* encodes another DEG/ENaC family sodium channel subunit that functions as a critical regulator of locomotion [40,41]. *trp-4* encodes a transient receptor potential (TRP) channel expressed in dopaminergic neurons and a number of other neurons [42]. *deg-3* encodes a ligand-gated calcium channel belonging to the nicotinic acetylcholine receptor family [43]. Dominant mutations in these genes induces necrosis of neurons: the *deg-1(u38)* mutation causes necrosis of the IL1 sensory neurons and the AVD, AVG, and PVC interneurons [44]; the *unc-8(n491sd)* mutation induces the necrosis of a number of motor neurons at L1 stage [45]; the *trp-4(ot337)* mutation results in the necrosis of dopaminergic neurons and a few non-dopaminergic neurons, including DVA and DVC, two mechanosensory neurons in the tail [31]; and the *deg-3(u662)* mutation triggers the necrosis of a number of sensory neurons including the six touch neurons, IL1, and PVD, and interneurons AVG and PVC [43]. *deg-1(u38)*, *unc-8(n491sd)*, *trp-4(ot337)*, and *deg-3(u662)* mutants all exhibited strong PS signal on the surfaces of necrotic cells (Fig 1), indicating that PS exposure is a common event occurring on necrotic sensory neurons, interneurons, and motor neurons of different identities and induced to undergo necrosis by mutations of different genes.

### A robust and transient increase in the cytoplasmic Ca^2+^ level is detected preceding PS exposure on necrotic neurons

**Fig 2:**
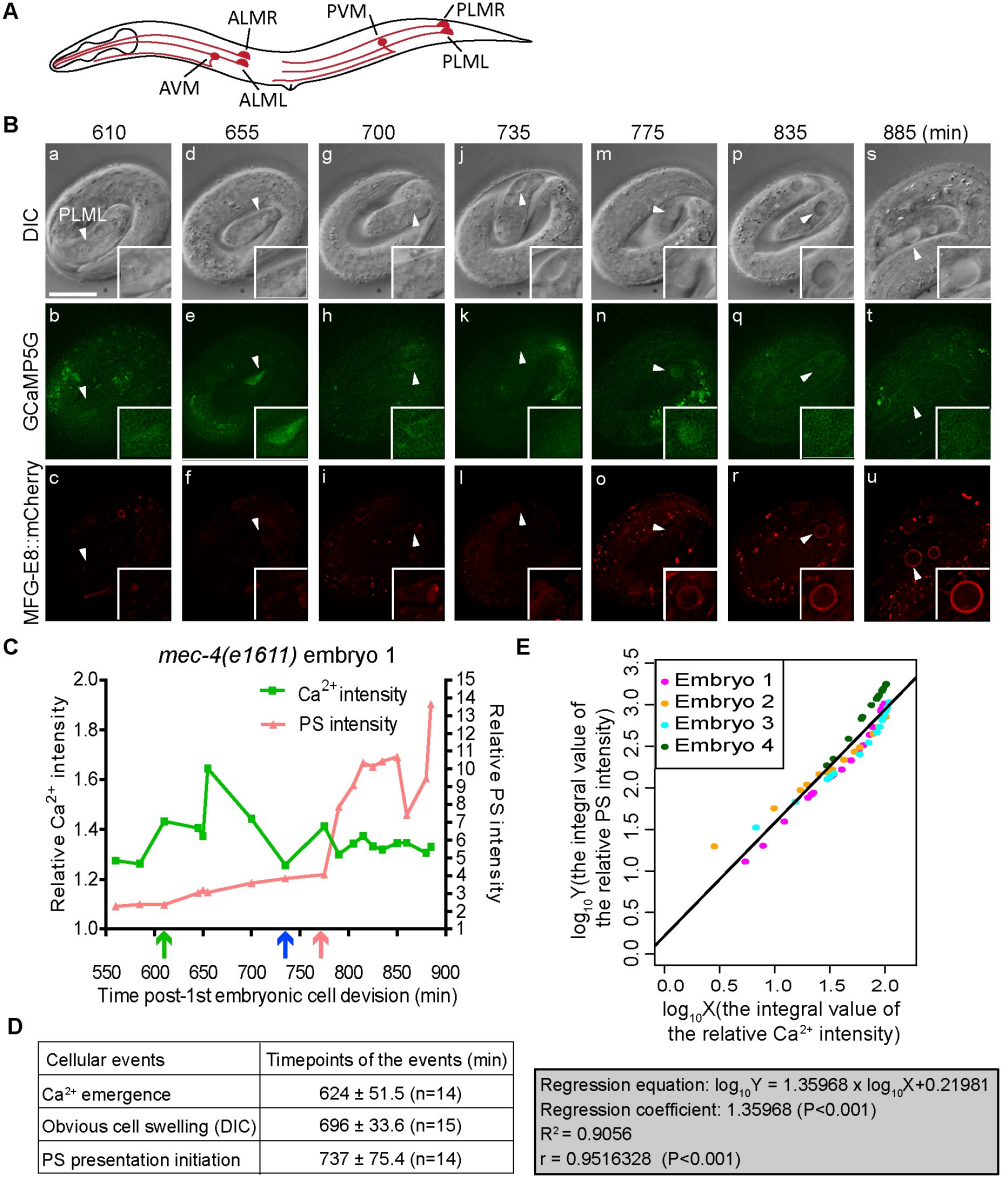
The cytoplasmic Ca^2+^ signal increases prior to the appearance of necrosis morphology and the exposure of PS, and displays a linear correlation with the PS level. Reported here are results of time-lapse recording experiments monitoring cytoplasmic Ca^2+^ levels, cell swelling, and PS signal in or on the surfaces of PLML and PLMR neurons. Embryos are *mec-4(e1611)* homozygotes carrying the *enIs92* transgenic array co-expressing P_*mec-7*_GCaMP5G and P_*dyn-1*_*mfg-e8*::*mCherry*. (A) Diagram of a hermaphrodite indicating the positions and names of the six touch neurons. (B) Time-lapse images monitoring the necrosis of one PLML neuron (white arrowheads). Time points are marked as min post-first embryonic cell division. Recording started at 100 min after the embryo reached 2-fold stage, at 560 min, and ended at 900 min. The GCaMP5G signal first appears in (b) and reaches its peak value in (e). PLML starts swelling in (g) and continues swelling in (j, m, p, s). The MFG-E8::mCherry signal on the surface of PLML becomes visible in (o) and increases over time (r, u). The scale bar is 10μm. Additional time-lapse recording for three more PLM neurons are shown in S1 Fig. (C) Relative signal levels (in comparison to the background levels) of GCaMP5G in the cytoplasm, and of MFG-E8::GFP on the membrane surface, of the PLML shown in (A). The green, blue, and red arrows underneath the X-axis mark the time points when the rise of Ca^2+^ signal, the distinct cell swelling morphology becomes obvious, and PS is first seen on the surface of the neuron, respectively, by eye observation. (D) Summary of the time points when each of the three cellular events in PLM neurons during the onset of necrosis: the appearance of Ca^2+^ in the cytoplasm (GCaMP5G), the distinct cell swelling morphology observed under DIC microscope, and the appearance of PS on the surface (MFG-E8::mCherry). Data represent mean ± standard deviation. n, the numbers of PLM neurons analyzed. (E) Linear regression analysis between the log-transformed relative cytoplasmic Ca^2+^ levels inside the PLM neurons and relative PS levels on the surfaces of the same neurons. The integral values of the Ca^2+^ and PS signals throughout the observed time period (560 min to 900 min post-1^st^ embryonic division) were calculated. Linear regression and Pearson’s correlation coefficient analysis were performed by the R Software. The plot was derived from time points from 4 independent time-lapse recording series (Fig 2B and S1A–S1C Fig). The data of different series are labeled in different colors. The data include 60 time points (18 from embryo 1, 14 each from embryos 2–4). X: the integral value of the relative Ca^2+^ intensity; Y: the integral value of the relative PS intensity; R^2^: coefficient of determination; r: correlation coefficient.

To determine whether cytoplasmic Ca^2+^ level changes as predicted during the necrosis of touch neurons [35], we constructed a Ca^2+^ indicator (GCaMP5G) that is expressed in touch neurons under the control of the P_*mec-7*_ promoter [46] and introduced P_*mec-7*_ *GCaMP5G* into the *mec-4(e1611)* mutant strain (Materials and methods). In the absence of Ca^2+^, GCaMP5G only emits a very low background level of fluorescence; in the presence of Ca^2+^, due to the conformation change of this fusion protein induced by its interaction with Ca^2+^, GCaMP5G generates a detectable GFP signal [47]. The P_*mec-7*_ *GCaMP5G* reporter is designed to detect cytoplasmic Ca^2+^ but not the Ca^2+^ inside the ER lumen because it does not have any ER signal sequence. We co-expressed GCaMP5G with the PS reporter MFG-E8::mCherry and established a time-lapse recording protocol that allowed us to simultaneously monitor Ca^2+^ and PS signals throughout embryonic development (Materials and methods). In *mec-4(e1611)* mutants, touch neurons PLML and PLMR (Fig 2A) undergo necrosis, evident by cell swelling (Figs 2B and S1E), during the late embryonic developmental stage [19]. We monitored throughout the necrosis process of PLML and PLMR and detected robust and transient increases of GCaMP5G intensity in the cytoplasm of the PLM neurons in the form of peaks that gradually reduced to the basal level (Figs 2B, 2D, 4B, S1A–S1C, and S2C). In all cases (Figs 2, 4B, S1A–S1C, and S2C), multiple peaks were observed, and at least one peak in each cell occurs prior to cell swelling. PS exposure occurs after cell swelling; and immediately prior to the detection of PS, there are additional transient Ca^2+^ peaks occurring (Figs 2B–2D and S1A–S1C). These observations demonstrate that the rise in cytoplasmic Ca^2+^ level precedes cell swelling and PS exposure. Together, the recording of multiple samples revealed that although there were broad ranges of variation of both the peak Ca^2+^ signal value and the width of the peak, a general pattern of cytoplasmic Ca^2+^ peaks preceding cell swelling and PS exposure remained consistent among necrotic neurons (Figs 2C, 4, S1, and S2C).

On the contrary, in live PLM neurons in the *mec-4(+)* embryos, only weak, background levels of GCaMP5G signal were observed (S2A and S2B Fig). Although the GCaMP5G signal also fluctuates, both the peak and basal values are much lower than in the necrotic PLM neurons in the *mec-4(e1611)* embryos, so are the duration period of the increased signal. We further calculated the integral Ca^2+^ signal levels over two periods: the first period covers from 560 min to 695 min post-1^st^ embryonic division, the mean time point that cell swelling is visible; the 2^nd^ period covers 696–800 min post-1^st^ embryonic division, during which PS signal starts to be detectable on the surfaces of necrotic cells and its intensity continues to increase (S1F Fig). We found that in *mec-4(+)* embryos, the mean Ca^2+^ integral values in periods 1 and 2 were 52.6% and 30.7% of that observed from necrotic PLM neurons in *mec-4(e1611)* mutants (S1D Fig). The low peak value, short duration period of the peak, and the overall low integral value (40.4% of that of *mec-4(e1611)* mutants) (S1D Fig) of the cytoplasmic Ca^2+^ signal together correlate with the lack of necrosis and PS exposure in live PLM neurons in wild-type embryos.

The above observations demonstrate that the cytoplasmic Ca^2+^ exhibits a unique and dynamic pattern of fluctuation in necrotic neurons that is not observed in their living counterparts. To examine the potential correlation between the signal levels of cytoplasmic Ca^2+^ and PS on the cell surface, in addtion to the integral values of Ca^2+^, we quantified the integral values of the relative signal intensities of PS over time. We subsequently conducted a linear-regression analysis between the log-transformed integral values of the cytoplasmic Ca^2+^ and cell-surface PS levels. Remarkably, we identified a very strong positive correlation between these two values (the correlation coefficient (r) is about 0.96) (Fig 2E). Together, the above observations and analyses suggest that the cytoplasmic Ca^2+^ makes a strong contribution to the level of PS exposure on the surfaces of necrotic neurons. Both the fold of increase of the Ca^2+^ intensity and the duration of the increased intensity are likely important factors for the activation of PS exposure machinery.

### Suppression of the release of Ca^2+^ from the ER by dantrolene represses the exposure of PS on necrotic neurons

**Fig 3:**
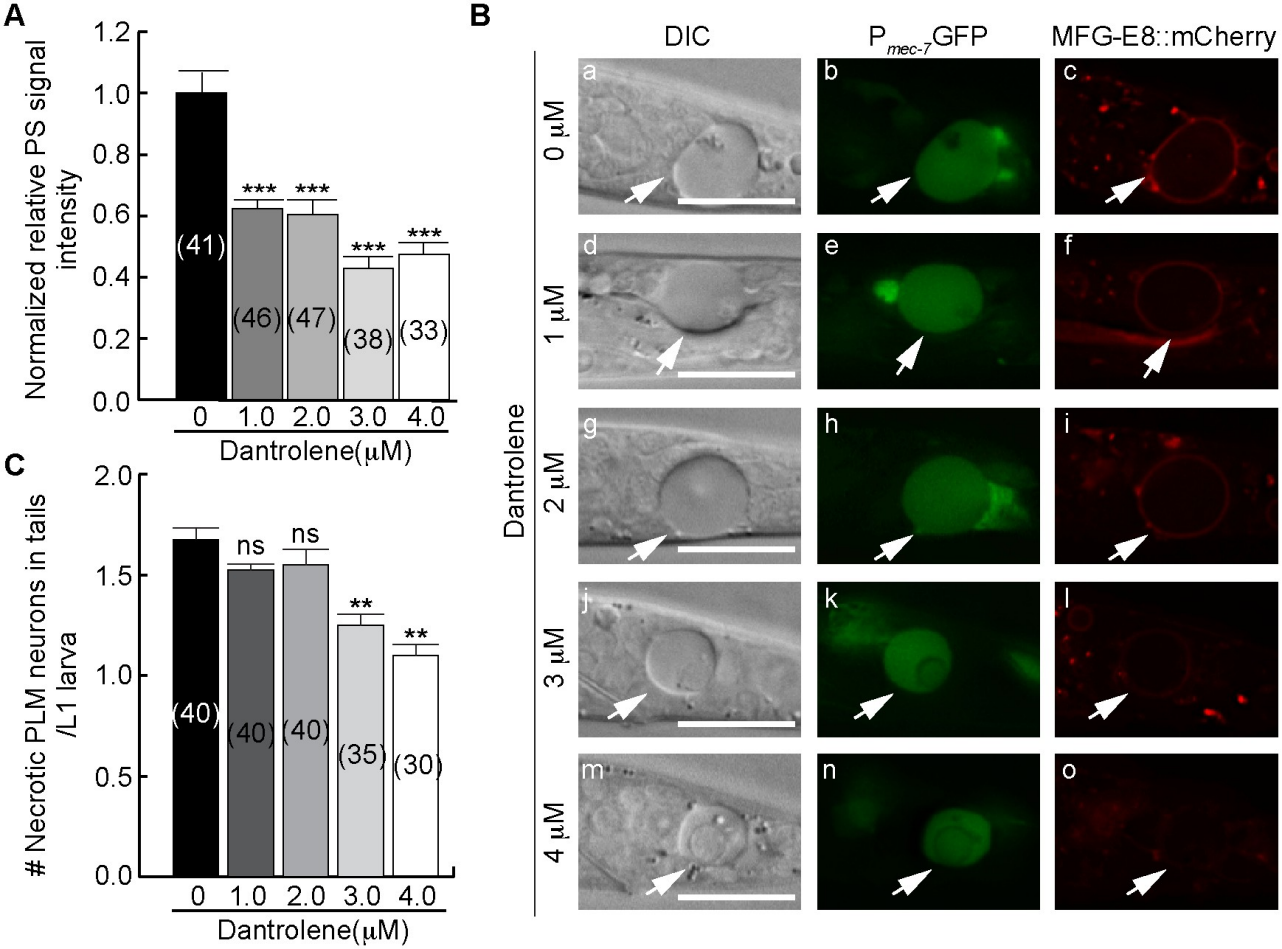
Dantrolene inhibits the exposure of PS on the surface of necrotic neurons. (A) The normalized relative PS intensity (normalized PS_R_) of necrotic PLMs in the tails of newly hatched *ced-1(e1735); mec-4(e1611)* L1 larvae. Adult worms were placed on NGM plates with different doses of dantrolene and allowed to lay eggs; shortly after hatching, larvae were scored. The “0 μM” samples were given DMSO with no dissolved dantrolene. Data are presented as mean ± standard error of the mean (s.e.m.). Numbers of necrotic PLM neurons scored for each dantrolene concentration are in paratheses. ***, p < 0.001, Student *t*-test. (B) DIC (a, d, g, j, m), P_*mec-7*_GFP (b, e, h, k, n), and MFG-E8::mCherry (c, f, i, l, o) images of the necrotic PLM neurons (arrows) in the newly hatched L1 larvae. The mother worms were incubated with different doses of dantrolene as labeled. PLM neurons are labeled with P_*mec-7*_GFP. Scale bars are 15 μm. (C) The mean numbers of necrotic PLM neurons in the tails of newly hatched L1 larvae generated by adult worms incubated with different doses of dantrolene. Data are presented as mean ± s.e.m. The numbers of L1 larvae scored after dantrolene treatments are in parentheses. **, 0.001 < p < 0.01; ns, no significant difference, Student *t*-test.

To determine whether the exposure of PS on necrotic touch neurons requires the release of Ca^2+^ from the ER, we examined whether blocking the release of Ca^2+^ from the ER in the *mec-4(e1611)* mutants would affect PS exposure on necrotic neurons. The ryanodine receptor (RyR) and InsP_3_ receptor (InsP_3_R) are two major kinds of Ca^2+^ release channels on the ER membrane [38]. Dantrolene, a small molecule antagonist of ryanodine receptors [48], was reported to suppress *mec-4(d)* induced necrosis in touch neurons [35]. We placed hermaphrodites that carried homozygous *mec-4(e1611)* alleles on NGM plates containing different doses of dantrolene (Materials and methods), and measured the cell surface PS intensity on the PLML and PLMR touch neurons in their progeny as newly hatched L1 larvae using MFG-E8::mCherry (Fig 3).

In every *mec-4(e1611)* mutant embryo, both the PLML and PLMR neurons undergo necrosis [19]. In worms that exhibit normal clearance of dying cells, these necrotic neurons are rapidly engulfed and degraded; as a consequence, some of them disappear before the embryo hatches [19,49]. To allow necrotic cells to persist during the larval stages, we included a *ced-1(e1735)* null mutation, which perturbs necrotic cell clearance [19], in the strains to be tested. In each *ced-1(e1735); mec-4(e1611)* double mutant L1 larva hatched within 1 hr, on average 1.7 instead of 2 necrotic PLMs were observed (Fig 3C), due to the residual cell corpse clearance activity that is still present in *ced-1* mutants [50,51]. We found that low doses of dantrolene (e.g. 1.0 and 2.0 μM) did not significantly affect the number of PLM neurons that undergo necrosis (Fig 3C), yet resulted in a ~40% reduction of PS level on the surface of necrotic PLMs (Fig 3A and 3B (d-i)). These results suggest that a modest level of inhibition of the release of Ca^2+^ from the ER affects the exposure of PS on necrotic touch neurons without significantly inhibiting necrosis. At higher doses (e.g. 3.0 and 4.0 μM), dantrolene resulted in 50% reduction of the PS intensity (Fig 3A and 3B) and significant reduction of necrosis events (Fig 3C). Collectively, these results suggest that while the Ca^2+^ released from the ER facilitates both necrosis and PS exposure, and PS exposure requires the support of a higher level of cytoplasmic Ca^2+^ than cell swelling does. Moreover, they imply that cytoplasmic Ca^2+^ might directly induce PS exposure independently of cell swelling.

**Fig 4:**
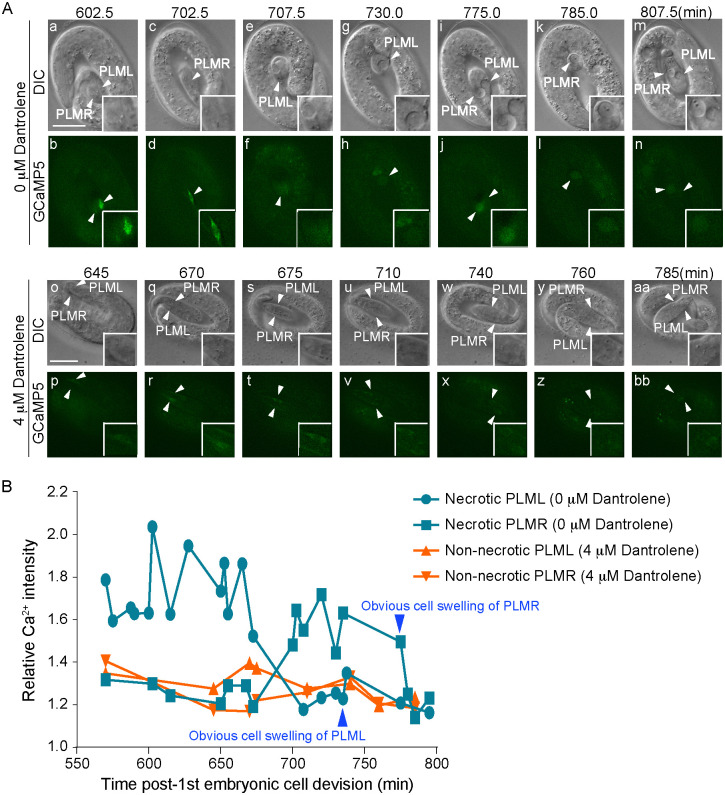
Dantrolene reduces the level of Ca^2+^ in the cytoplasm of touch neurons. Shown here are results of time-lapse recording experiments measuring the intensity of cytoplasmic Ca^2+^ and cell swelling of PLM neurons over time. The strain is homozygous for *mec-4(e1611)* allele and carries a transgenic array expressing P_*mec-7*_ *GCaMP5G*::*gfp*. (A) Time-lapse recording images of two necrotic PLM neurons in one untreated embryo (a-n) and two living PLM neurons in one embryo from the 4μM dantrolene treated adult (o-bb). (a-n) Because PLML and PLMR do not appear in the same focal planes, at every time point only one of the two (identity labeled) is visible. (o-bb) No PLM swelling was observed in this embryo. Scale bars are 10μM. (B) The relative fluorescence intensities of cytoplasmic GCaMP5 in the PLM neurons shown in (A) are plotted over time. Arrowheads mark the time points when cell swelling became distinct in the 0μM dantrolene samples. The two 4μM dantrolene samples did not display cell swelling morphology.

To examine whether the dantrolene treatment indeed reduces the concentration of cytoplasmic Ca^2+^ in the PLM neurons in *mec-4(e1611)* mutant embryos, we monitored the GCaMP5G reporter over time. We first measured the Ca^2+^ levels in PLMs whose necrosis was suppressed by 4 μM dantrolene and thus remained living and compared them to their untreated counterparts, and found that dantrolene treatment greatly reduced the Ca^2+^ levels in the cytoplasm of PLM neurons throughout embryonic development (Figs 4 and S2C). Remarkably, dantrolene treatment disrupted the transient cytoplasmic Ca^2+^ peak characteristic of necrosis (Fig 4A (o-bb)). Secondly, we compared the cytoplasmic Ca^2+^ levels in necrotic PLM neurons in worms either through 3 μM dantrolene treatment or not treated with dantrolene. After 3 μM dantrolene treatment, the GCaMP5G signal intensity in necrotic PLM neurons display peaks that are lower and more ephemeral than their untreated counterparts (S2C and S2D Fig). Together, the heavily suppressed cytoplasmic Ca^2+^ signal patterns demonstrate that dantrolene treatments indeed cause the reduction of cytoplasmic Ca^2+.^ in the *mec-4(d)* background. These patterns explain the reduced PS level on the surface of necrotic PLM neurons and the partial suppression of necrosis, which is represented by cell swelling (Fig 3).

### Impairing the establishment of the Ca^2+^ pool in the ER inhibits PS exposure on necrotic neurons

To further dertermine the role of the ER Ca^2+^ pool in inducing PS exposure, we analyzed the *crt-1*(*lf*) mutants in which the establishment of Ca^2+^ pool in the ER is defective. *crt-1* encodes the only *C*. *elegans* homolog of mammalian calreticulin, an ER-residential calcium chaperon that binds free Ca^2+^ and enables the accumulation of Ca^2+^ in the ER [52]. Calreticulin-deficient cells are defective in the storage of Ca^2+^ in ER [53]. In *C*. *elegans*, the *crt-1(bz29)* null mutation ([35] and Materials and methods) fully suppressed the necrosis of touch neurons induced by *mec-4(e1611)* mutation (0 PLM neuron underwent necrosis among 1000 L1 larvae scored), supporting the notion that in *crt-1(bz29)* mutants, the ER fails to release sufficient amount of Ca^2+^ to the cytoplasm [35]. We measured the level of cytoplasmic Ca^2+^ in *crt-1(bz29); mec-4(e1611)* double mutant embryos carrying the GCaMP5G reporter and found that in four PLM neurons whose cell-swelling morphology was suppressed, the cytoplasmic Ca^2+^ level remained low throughout the recording period from mid-embryogenesis to hatching (S3 Fig). Even when a small and transient peak of signal was observed, the peak value never exceeded 1.2-fold of the background signal value (S3A and S3B Fig). In comparison, in *crt-1(+); mec-4(e1611)* strain, the peak Ca^2+^ signal levels vary from 1.4- to 5-fold of the background level (Figs 2B–2C and S1A–S1C). The integral Ca^2+^ intensity values in *crt-1(bz29); mec-4(e1611)* embryos for the entire time-lapse recording period (560–800 min post 1^st^ embryonic division) and for the early (560–695 min) and late (696–800 min) periods, respectively, are less than 25%, 35%, and 30% of that in *mec-4(e1611)* embryos, respectively (S3C Fig). The low level of cytoplasmic Ca^2+^ suggests a failure of the ER to release Ca^2+^ to the cytoplasm and further implies that the ER Ca^2+^ pool is not properly established in *crt-1* null mutant background.

**Fig 5:**
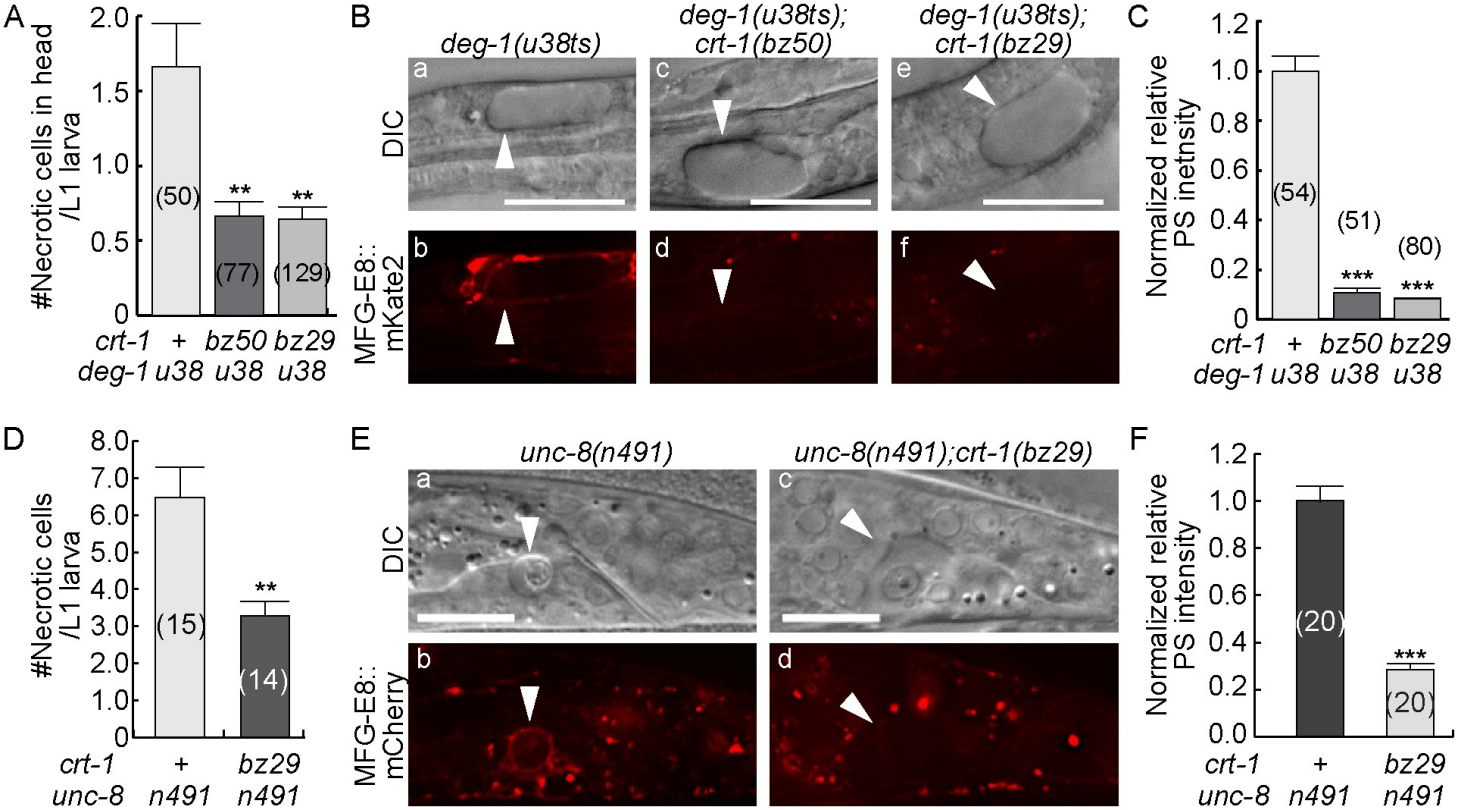
Mutations in *crt-1* inhibit the exposure of PS on the surface of necrotic cells in *deg-1* and *unc-8* dominant mutant animals. Newly hatched L1 larvae of labeled genotypes expressing the PS reporter P_*ced-1*_*mfg-e8*::*mKate2* or P_*ced-1*_*mfg-e8*::*mCherry* are scored for the the number of necrotic cells (A and D) and the relative PS intensity (C and F). All strains carry the *deg-1(u38ts)* allele were incubated at 25°C. (A and D) The mean numbers of necrotic cells in the head of each newly hatched larva (A) or in the entire body of a late L1/early L2 stage larva (D) are represented as bars. Error bars represent s.e.m.. Numbers in parentheses indicate the number of animals analyzed for each strain. “**”, <0.001<p<0.01, Student *t*-test. (B) DIC (a, c, e) and mKate2 (b, d, f) images of necrotic cells (arrowheads) in the heads of L1 larvae. Scale bars are 15μm. (E) DIC (a, c) and mCherry (b, d) images of necrotic cells (arrowheads) in the tails of ealy L2 larvae. Scale bars are 10μm. (C and F) The normalized relative signal intensities of PS on the surfaces of necrotic cells in the heads (C) or tails (F) are presented as the mean ratios relative to *crt-1(+)* worms. Bars represent the mean values of each strain. Error bars represent s.e.m.. Numbers in parentheses represent the number of necrotic cells analyzed. “***”, p<0.001, Student *t*-test.

Two *crt-1* mutations, the *bz29* null mutation and *bz50*, another loss-of-function mutation [35], partially suppress necrosis in *deg-1(u38ts)* mutants and reduce the number of necrotic cells (Fig 5A) [35]. In *crt-1(lf); deg-1(u38ts)* double mutants and *deg-1(u38ts)* single mutant worms, we examined PS exposure on necrotic neurons using P_*ced-1*_*mfg-e8*::*mKate2* (Materials and methods). In *deg-1(u38ts)* mutants, a small number of sensory and interneurons undergo necrosis at 25°C, the restrictive temperature [44] (Fig 5A), and expose PS on their surfaces (Fig 5B (b)). In both *crt-1* alleles, the levels of PS signal on the existing necrotic cells is reduced to <10% of that in the *crt-1(+)* animals (Fig 5B (c-f) and 5C). The *crt-1* mutation also partially suppresses necrosis in the *unc-8(n491)* background (Fig 5D). In the *unc-8(n491); crt-1(bz29)* double mutants (Materials and methods), PS signal intensity is reduced to on average 29% of that of the *unc-8* single mutants (Fig 5E and 5F). Together, the above results strongly suggest that a normal ER pool of Ca^2+^ and the release of Ca^2+^ into the cytoplasm are essential for proper PS exposure on cells induced to undergo necrosis by hyperactive mutations in subunits of the DEG/ENaC channels.

### Ectopically increasing cytoplasmic Ca^2+^ induces necrosis and the exposure of PS

**Fig 6:**
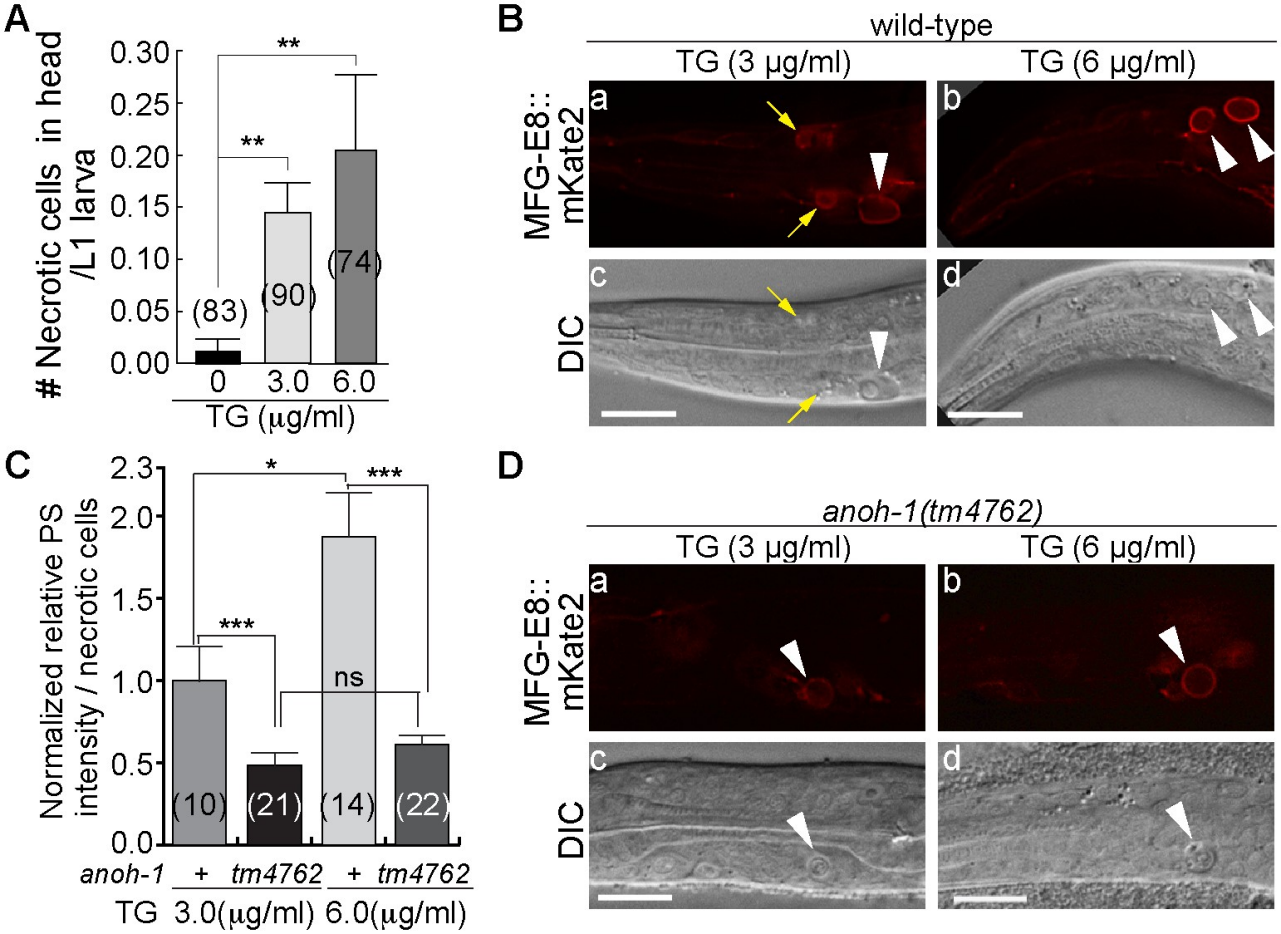
Thapsigargin induces living cells to undergo necrosis and expose PS. Adult worms carrying the integrated PS reporter P_*ced-1*_ *mfg-e8*::*mKate2* were placed on NGM plates with different doses of thapsigargin (TG) and incubated at 25°C; their progeny were allowed to hatch into L1 larvae and promptly scored for the number of necrotic corpses in the head (A) and the intensity of PS on the surface of necrotic cells (B-D). (A) The mean numbers of necrotic cells in each wild-type L1 larva are represented in the bar graph. Error bars represent s.e.m.. “**”, 0.001<p<0.01, Student *t*-test. (B and D) MFG-E8::mKate2 (a, b) fluorescence and DIC (c, d) images of necrotic cells (white arrowheads) observed in the heads of wild-type (B) and *anoh-1* mutant (D) larvae treated with differet doses of TG. Small yellow arrows in (a, c) mark two small, necrotic-like cells (possibly intermediate necrotic cells) that expose PS. Because of their small sizes, they were not counted as necrotic cells. Scale bars are 10μm. (C) The normalized relative MFG-E8::mKate2 signal intensities on the surfaces of necrotic cells in the heads of wild-type and *anoh-1(tm4762)* mutant L1 larvae are presented as the mean ratios relative that measured from worms subject to 3 μM thapsigargin treatment. Genotypes are indicated underneath the X-axis. Error bars represent s.e.m.. Numbers in parentheses represent the number of animals analyzed for each strain. “***”, p<0.001, “*”, 0.01<p<0.05, “ns”, non-sigificant, Student *t*-test.

If the level of cytoplasmic Ca^2+^ determines whether a cell exposes PS and undergoes necrosis or not, presumably an ectopic increase of cytoplasmic Ca^2+^ level should induce cells to expose PS. To test this possibility, we treated wild-type worms with thapsigargin (Materials and methods). Thapsigargin inhibits Sarco-Endoplasmic Reticulum Ca^2+^ ATPase (SERCA), a Ca^2+^ re-uptake pump located on the ER and sarcoplasmic reticulum (SR) that pumps Ca^2+^ from the cytoplasm to the ER and SR [54,55]. Inhibiting SERCA activity increases the level of cytoplasmic Ca^2+^ [55]. Thapsigargin treatment was reported to induce PS exposure on the surfaces of platelets [56]. We observed that, consistent with the previously report [35], thapsigargin treatment induced the onset of necrosis of cells that are otherwise destined to live in the head (Fig 6A and 6B), whereas untreated worms exhibited nearly no necrotic cells (only one necrotic cell observed in a popuation of 83 larvae) (Fig 6A). In addition, thapsigargin-induced necrotic cells expose PS on their surfaces; furthermore, a higher concentration of thapsigargin correlates with stronger PS exposure (6 μg/ml vs 3 μg/ml thapsigargin) (Fig 6B and 6C). These results support a model proposing that the PS exposure on cell surfaces is dependent on the concentration of cytoplasmic Ca^2+^.

### Induction of PS exposure is independent of other necrosis features

Our observation reported in Fig 3 implies that cytoplasmic Ca^2+^ might induce cell swelling and PS exposure by targeting independent effectors. We reason that if PS exposure is a consequence of the execution of necrosis, blocking the intermediate steps of necrosis execution would inhibit PS exposure even when cytoplasmic Ca^2+^ level is increased via ER-mediated Ca^2+^ release; otherwise, PS exposure will not be blocked but other necrotic cell events such as cell swelling will. We applied two different methods to impair the intermediate necrosis events, and examined whether PS exposure is affected in each case.

**Fig 7:**
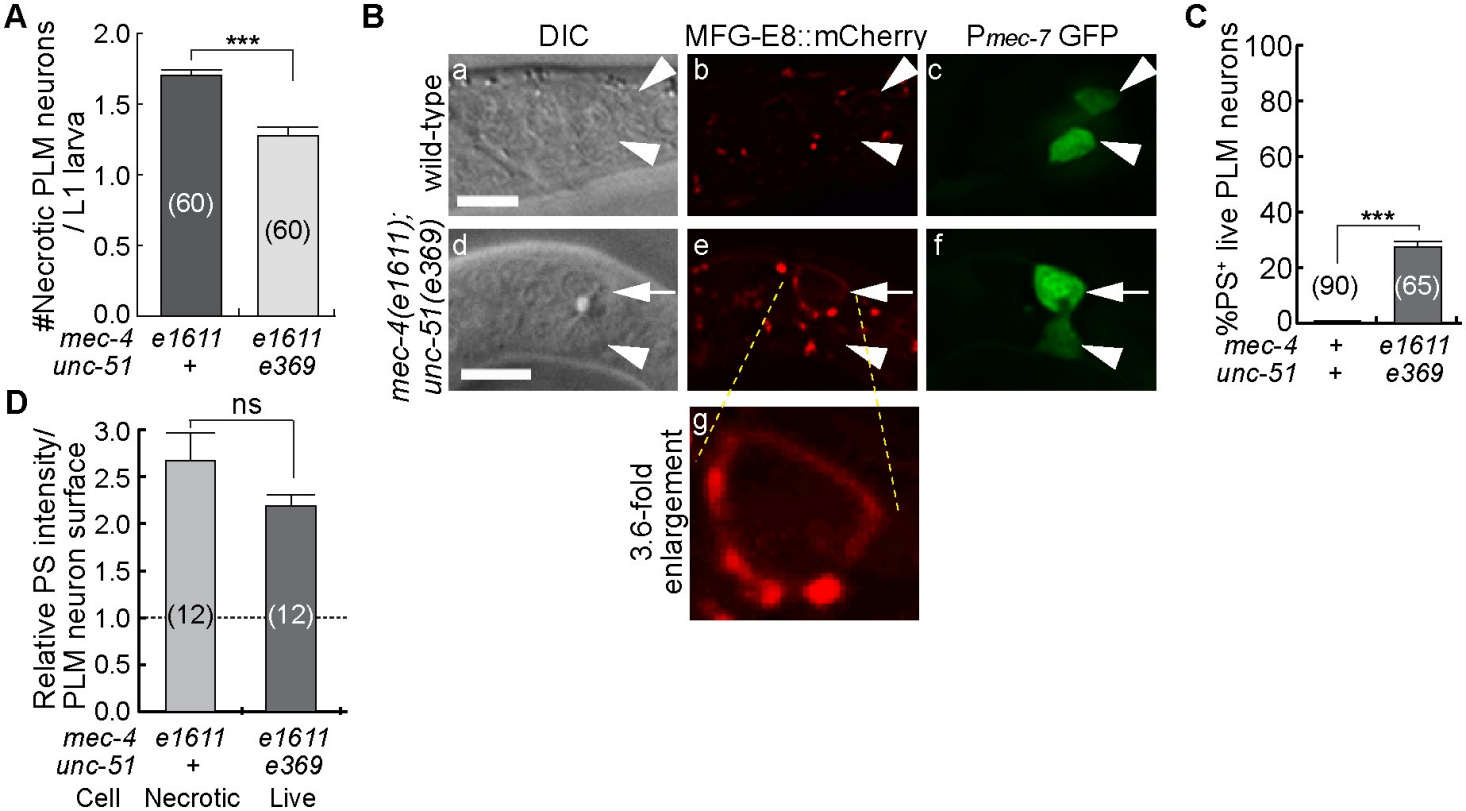
Impairing the execution of necrosis does not block PS exposure. (A) The mean number of PLM neurons (labeled with the P_*mec-7*_ GFP reporter) in *ced-1(e1735); mec-4(e1611)* and *ced-1(e1735); unc-51(e369); mec-4(e1611)* L1 larvae that displayed the necrotic swelling phenotype were presented in the graph. L1 larvae were scored within 1 hr of hatching. Bars represent the mean values of each sample. Error bars represent s.e.m.. The numbers in the parentheses represent the numbers of animals scored. “***”, p<0.001, Student *t*-test. (B) DIC, GFP, and mCherry images of four living PLM neurons, two in a wild-type L1 larva (a-c, white arrowheads), and two in a *ced-1(e1735); unc-51(e369); mec-4(e1611)* L1 larva (d-f, white arrows and arrowheads). PLM neurons are labeled with the P_*mec-7*_GFP reporter (c, f). DIC images (a, d) show that none of the four PLM neurons display the necrotic swelling morphology. PS presentation (MFG-E8::mCherry) on the surface of one PLM neuron (e) is marked by a white arrow (e). White arrowheads in (a-f) mark living PLM neurons that do not expose PS. Scale bars are 5μm. (g) 3.6-fold enlarged image of the region where the PS^+^ living cell (e, arrow) is. (C) A bar graph representing the percentage of live PLM neurons that expose PS on their surfaces among all living PLM neurons in early L1 larvae with the indicated genotypes. Error bars represent s.e.m.. The numbers in the parentheses represent the total numbers of living PLMs. “***”, p<0.001, Student *t*-test. (D) Relative PS intensity on the surfaces of PLM neurons in newly hatched L1 larvae. In *mec-4(e1611)* mutants, necrotic PLM neurons were measured. In *unc-51(e369); mec-4(e1611)* mutants, living PS^+^ PLM neurons were measured. Data are presented by mean ± sem. ns, non-significant, Student t-test.

We first explored the effect of an *unc-51(e369)* mutation on PS exposure. *unc-51* encodes a homolog of yeast ATG1 and mammalian ULK1 (UNC-51-like autophagy activating kinase), an important component of the autophagy pathway [57]. Autophagy genes actively participate in the lysosomal-dependent necrosis, which causes cell swelling and other cellular events [58,59]. Specifically, a loss-of-function mutation of *unc-51* was reported to partially block the execution of necrosis induced by a *mec-4(d)* allele [58,59]. We introduced the *unc-51(e369) lf* allele into the *ced-1(e1735); mec-4(e1611)* mutant strain that carries the MFG-E8::mCherry and P_*mec-7*_*gfp* reporters. We specifically monitored the PLML and PLMR neurons in the tail of young L1 larvae. Consistent with previous reports [58,59], we observed a significant and sizable reduction (25%) in the number of necrotic PLM neurons in newly hatched *ced-1(e1735); unc-51(e369); mec-4(e1611)* L1 larvae comparing to that observed in *ced-1(e1735); mec-4(e1611)* L1 larvae (Fig 7A). Remarkably, in *ced-1(e1735); unc-51(e369); mec-4(e1611)* L1 larvae, PS was detected on 27.4% of the PLM neurons (labeled with P_*mec-7*_ GFP) that appeared live and displayed a relative normal and non-swelling morphology under the DIC microscope (Fig 7B and 7C), whereas in *ced-1(e1735)*; *unc-51(+); mec-4(+)* L1 larvae in which all PLM neurons were alive, 0% of living PLM neurons exposed PS (Fig 7B and 7C). Fig 7B (d-g) displayed an example of an apparently living PLM neuron that exposed PS. This phenomenon reveals that PS exposure could be induced by the *mec-4(d)* mutation in a manner independent of necrotic cell swelling.

We next attempted to impair the execution of necrosis through disrupting lysosomal function. During excitotoxic necrosis, increased cytoplasmic concentration of Ca^2+^ is known to activate a cascade of events that lead to the breakage of lysosomes, the acidification of the entire cell, and the release of lysosomal hydrolytic enzymes into the cytoplasm [60,61]. Cell swelling is a primary consequence of these events [60,61]. To disrupt lysosomal function, we applied NH_4_Cl, a reagent that impairs lysosomal acidification and partially blocks the execution of necrosis of touch neurons in the *mec-4(d)* mutants [60], to a liquid worm culture (Materials & methods). In the *ced-1(e1735)*; *mec-4(e1611)* mutant animals, 5mM NH_4_Cl caused a 40% reduction in the necrosis of the PLM neurons in L1 larvae (S4A Fig), consistent with a previous report [60]. In an average of 5.0% of 78 live (non-swelling) PLM neurons analyzed, we observed PS exposure on their cell surfaces (S4B(d-f) and S4C Fig, white arrows). Together, the results obtained from the *unc-51* mutants and the NH_4_Cl experiment indicate that the exposure of PS is not dependent on the full execution of necrosis.

### In *deg-3* and *trp-4* mutants, the exposure of PS does not rely on the ER Ca^2+^ pool

**Fig 8:**
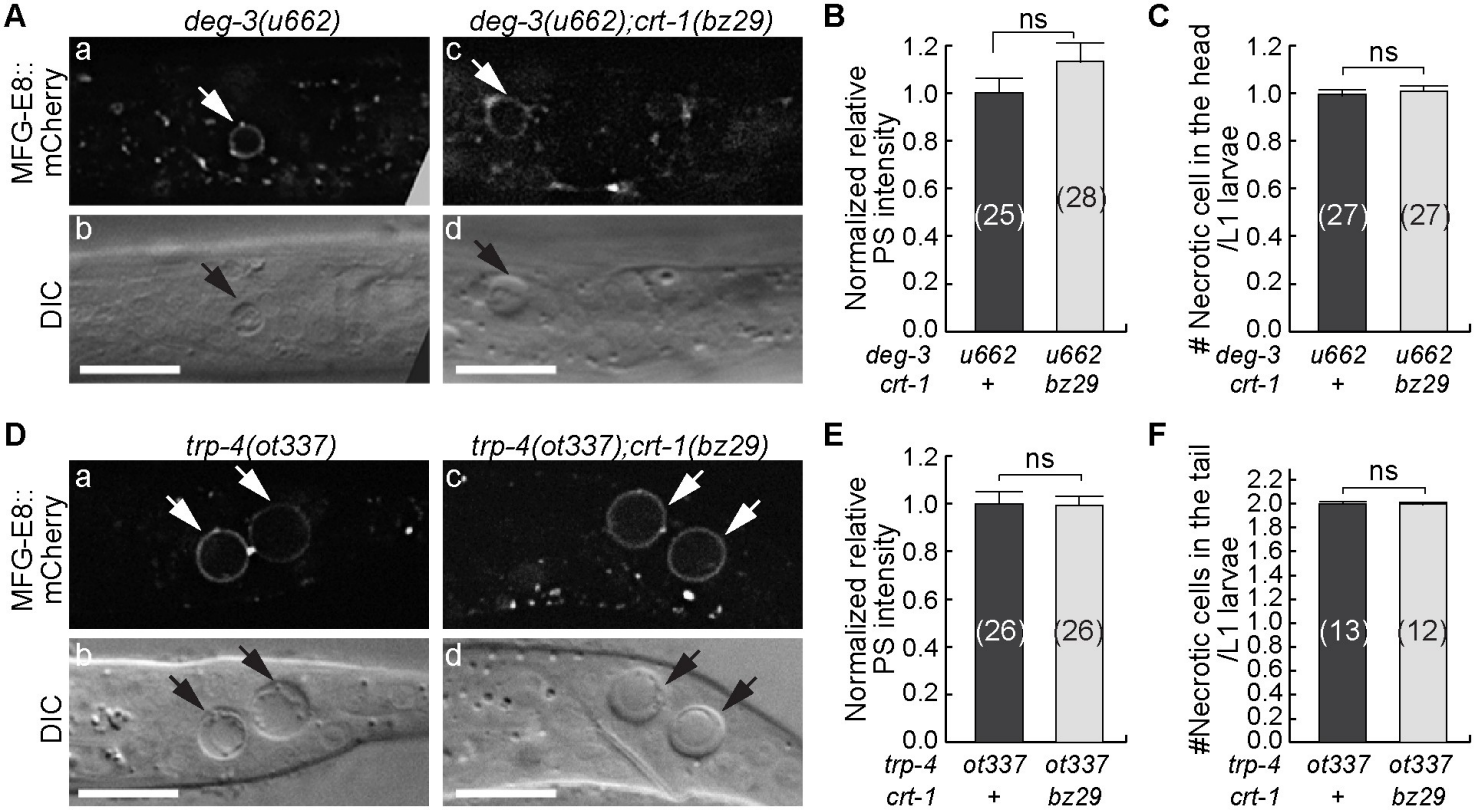
The *crt-1* null mutation does not affect the exposure of PS on necrotic cells in *deg-3* or *trp-4* mutants. (A) and (D) DIC (b, d) and MFG-E8::mCherry (a, c) images of necrotic cells (arrows) in the heads (A) and tails (D) of L1 larvae, respectively. Genotypes are labeled on the top. Scale bars are 10μm. (B) and (E) The normalized relative signal intensities of MFG-E8::mCherry on the surfaces of necrotic cells in the heads (B) or tails (E) of L1 larvae are presented as the mean ratios relative to *crt-1(+)* worms. Bars represent the mean values of each strain. Error bars represent s.e.m.. Numbers in parentheses represent the number of necrotic cells analyzed. Mutant alleles are listed underneath the X-axis. ns, no significant difference, Student *t*-test. (C) and (F) The mean numbers of necrotic cells in the heads (C) and tails (F) of each L1 larva are represented as bars. Error bars represent s.e.m.. Numbers in parentheses indicate the number of animals analyzed for each strain. Mutant alleles are listed underneath the X-axis. ns, no significant difference, Student *t*-test.

*deg-3* encodes a ligand-gated calcium channel belonging to the nicotinic acetylcholine receptor family [43]. *trp-4* encodes a transient receptor potential (TRP) channel belonging to the TRPN subfamily [42]. TRP channels are non-voltage gated Ca^2+^ channels that are also permeable to Na^+^, and K^+^ [62,63]. In *deg-3(u662)* and *trp-4(ot337)* dominant mutants, PS is observed on the surfaces of necrotic cells (Fig 1I–1L). We constructed *crt-1(bz29); deg-3(u662)* and *crt-1(bz29); trp-4(ot337)* double mutants that carry the MFG-E8::mCherry reporter by introducing the *crt-1(bz29)* mutation into the *crt-1* gene in the genome via CRISPR/Cas9 mediated gene editing (Materials and methods). Unlike in *crt-1(bz29); deg-1(u38)* or *crt-1(bz29); unc-8(n491)* double mutants (Fig 5), in *crt-1(bz29) deg-3(u662)* and *trp-4(ot337); crt-1(bz29)* double mutants, the relative PS intensities on necrotic cells are the same as in the *deg-3* and *trp-4* single mutants, respectively (Fig 8A, 8B, 8D, and 8E). These results indicate that in the cells induced to undergo necrosis by hyperactive Ca^2+^ channels, a normal Ca^2+^ pool in the ER appears not essential for PS exposure. Previously it was reported that the *crt-1(bz29)* mutation does not suppress the necrosis induced by *deg-3(u662)* [35]. We confirmed this result (Fig 8C). Xu et al [35] proposed that the *deg-3* dominant mutation results in the influx of ample amount of Ca^2+^ into the neurons so that the contribution of Ca^2+^ from the ER is not needed for the induction of necrosis. Similarly, the *crt-1(bz29)* mutation does not suppress the necrosis of neurons in the tail in *trp-4* mutant larvae (Fig 8F). Together, our results indicate that the hyperactive Ca^2+^ channel mutations induce PS exposure through an ER-independent mechanism.

### The Ca^2+^-induced PS exposure on necrotic cells is dependent on ANOH-1

The *anoh-1(tm4762) lf* mutation reduces the PS signal intensity on the surfaces of necrotic cells in the *mec-4(e1611)* mutants ([19] and see Introduction). To obtain direct evidence that the increase of cytoplasmic Ca^2+^ induces PS exposure through targeting ANOH-1, we tested whether the PS exposure induced by thapsigargin requires the function of ANOH-1. We found that in *anoh-1(tm4762)* mutant L1 larvae, the PS intensity on necrotic cells were reduced (Fig 6C and 6D). For example, after the treatment of 6μg/ml thapsigargin, the mean PS signal intensity displayed by *anoh-1* mutants is only 32.7% of that measured from the wild-type background (Fig 6C). These results indicate that ANOH-1 plays an important role in Ca^2+^-induced PS exposure on necrotic cells.

**Fig 9:**
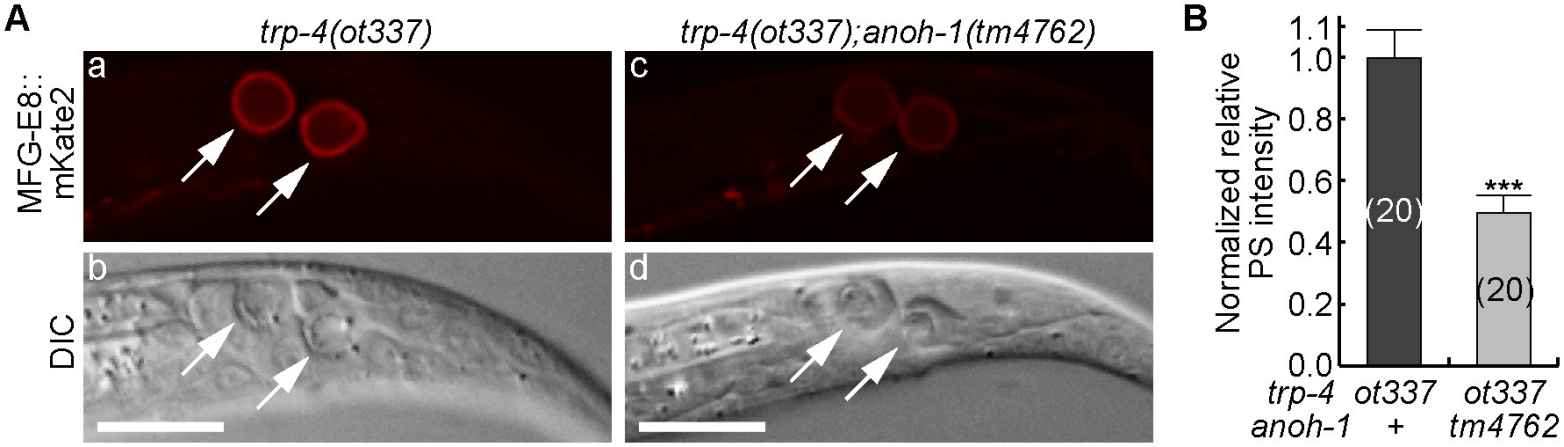
The *trp-4(ot337)*-induced PS exposure is dependent on the function of ANOH-1. (A) DIC (b, d) and MFG-E8::mKate2 (a, c) images of necrotic cells (arrows) in the tails of L1 larvae. Genotypes are labeled on the top. Scale bars are 10μm. (B) Bar graph of the normalized relative PS signal intensities on the surfaces of two necrotic cells in the tail of each *trp-4(ot337)* single mutant and *trp-4(ot337); anoh-1(tm4762)* double mutant L1 larva. Bars represent the mean values of each strain. Error bars represent s.e.m.. Numbers in parentheses represent the number of necrotic cells analyzed. Mutant alleles are listed underneath the X-axis. ***, p<0.001, Student *t*-test.

The *trp-4(ot337)*-induced PS exposure does not appear to require the ER release of Ca^2+^ (Fig 8D and 8E). We further tested whether ANOH-1 was needed for the exposure of PS on the two necrotic cells located in the tails of L1 larvae. We found that, in *trp-4(ot337); anoh-1(tm4762)* double mutants, the PS signal on the necrotic cells was reduced to 49% of that observed in *trp-4(ot337)* single mutants (Fig 9). This observation indicates that ANOH-1 function is also required for the ER-independent PS exposure pathway.

### Impairing the release of ER Ca^2+^ into the cytoplasm does not affect PS exposure on the surfaces of apoptotic cells

**Fig 10:**
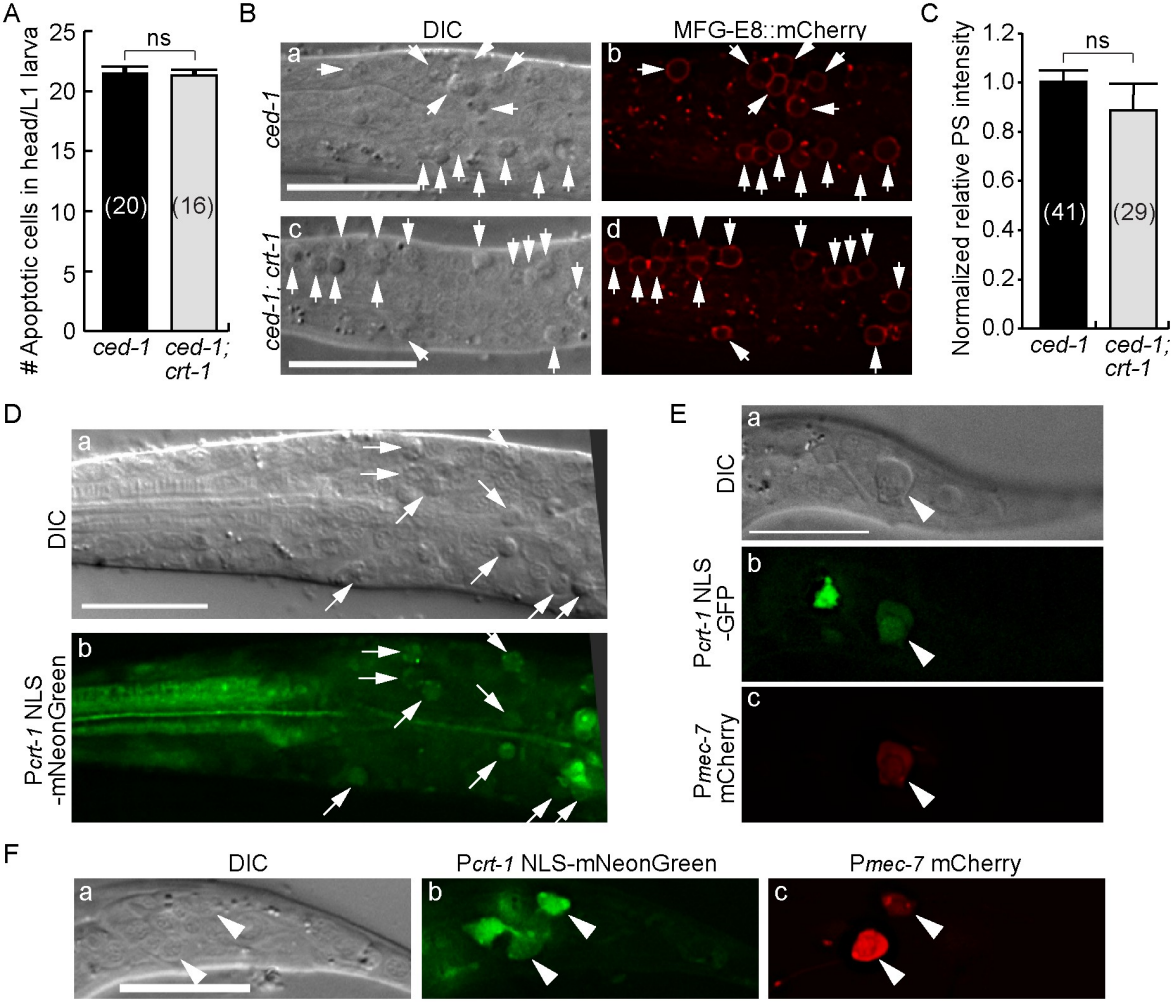
A null mutation in *crt-1* does not affect the exposure of PS on the surface of apoptotic cells. (A) In *ced-1(e1735)* and *ced-1(e1735); crt-1(bz29)* mutant strains, the number of apoptotic cells in the head of L1 larvae hatched within 1 hr were scored. Bars represent the mean values of each sample. Error bars represent s.e.m.. Numbers in parentheses represent the the numbers of L1 larvae scored. ns, statistically not significant (p>0.05, Student *t*-test). (B) DIC and fluorescence images of the heads of two larvae displaying numerous apoptotic cells (a and c, arrows). The PS signal exposed on their surfaces are detected by MFG-E8::mCherry (b and d, arrows). Scale bars are 10 μm. (C) Apopotic cells in the heads of larvae were scored for the normalized MFG-E8::mCherry signal intensity on their cell surfaces. The signal intensity was compared between two different mutant strains. Bars represent the mean values of each sample. Error bars represent s.e.m.. Numbers in parentheses are the the numbers of apoptotic cells analyzed. ns, statistically not significant (p>0.05, Student *t*-test). (D-F) The *crt-1* promoter is expressed in apoptotic cells (D), a necrotic touch neurons (E), and two living touch neurons (F). Transgenes were expressed in *ced-1(e1735); mec-4(e1611)* double (D and E) and *ced-1(e1735)* single (F) mutant strains. Scale bars are 15 μm. (D) DIC (a) and fluorescence (b) images of the head of an L2/L3-stage larva expressing P_*crt-1*_NLS-mNeonGreen. Arrows mark apoptotic cells. (E) DIC (a) and fluorescence (b and c) images of the tail of an L1-stage larva co-expressing P_*crt-1*_NLS-GFP (b) and the touch neuron marker P_*mec-7*_mCherry (c). Arrowheads mark one necrotic PLM neurons. (F) DIC (a) and fluorescence (b and c) images of the tail of an L1-stage larva co-expressing P_*crt-1*_NLS-GFP (b) and P_*mec-7*_mCherry (c). Arrowheads mark living PLM neurons.

During apoptosis in *C*. *elegans*, whether cytoplasmic Ca^2+^ contributes to the PS exposure on apoptotic cells was not known. To address this question, we examined whether a *crt-1(bz29)* null mutation, which disrupts the Ca^2+^ pool in the ER, would affect PS exposure on apoptotic cells. In the *ced-1(e1735)* mutants, the clearance of apoptotic cells is largely inhibited and many apoptotic cells persist in the head of newly hatched L1 larvae (Fig 10A and 10B). Loss of *ced-1* function does not affect PS dynamics on the surfaces of dying cells [19,20], and thus all of these apoptotic cells expose PS on their cell surfaces (Fig 10B and 10C). In the *ced-1(e1735); crt-1(bz29)* double mutant L1 larvae, the *crt-1(bz29)* null mutation does not affect either the number of apoptotic cells or the level of PS exposure on apoptotic cells (Fig 10A–10C).

*crt-1* is broadly expressed in *C*. *elegans* [35,64]. We next determined whether *crt-1* was expressed in cells destined to undergo apoptosis and/or necrosis. We constructed P_*crt-1*_*NLS-gfp* and P_*crt-1*_*NLS-mNeonGreen* reporters, in which the GFP or mNeonGreen reporters were tagged with a nuclear localization signal (NLS) and expressed under the direct control of the *crt-1* promoter (P_*crt-1*_) [64] (Materials & methods). The *crt-1* open reading frame is not present in these two reporters. The NLS sequence facilitates the enrichment of GFP or mNeonGreen in the nucleus. In the *ced-1(e1735); mec-4(e1611)* double mutant animals that expressed the P_*crt-*1_ *NLS-gfp* or P_*crt-*1_ *NLS-mNeonGreen* constructs, mNeonGreen and GFP signals were observed broadly, including in many apoptotic cells retained in the head (Fig 10D) as well as the necrotic touch neurons in the tail of L1 larvae (Fig 10E). In addition, in *ced-1(e1735)* single mutant larvae, P_*crt-1*_ NLS-mNeonGreen expression was observed in live PLM neurons in the tail (Fig 10F). These results indicate that *crt-1* is expressed in cells destined to die of apoptosis and necrosis. Therefore, collectively, our observations support the hypothesis that the ER Ca^2+^ pool does not regulate either the initiation of apoptosis or the exposure of PS on apoptotic cells, despite that *crt-1* is expressed and presumably functional in cells distined to undergo apoptosis. Our findings thus demonstrate that necrotic and apoptotic cells employ different molecular mechanisms to trigger PS exposure.

## Discussion

**Fig 11:**
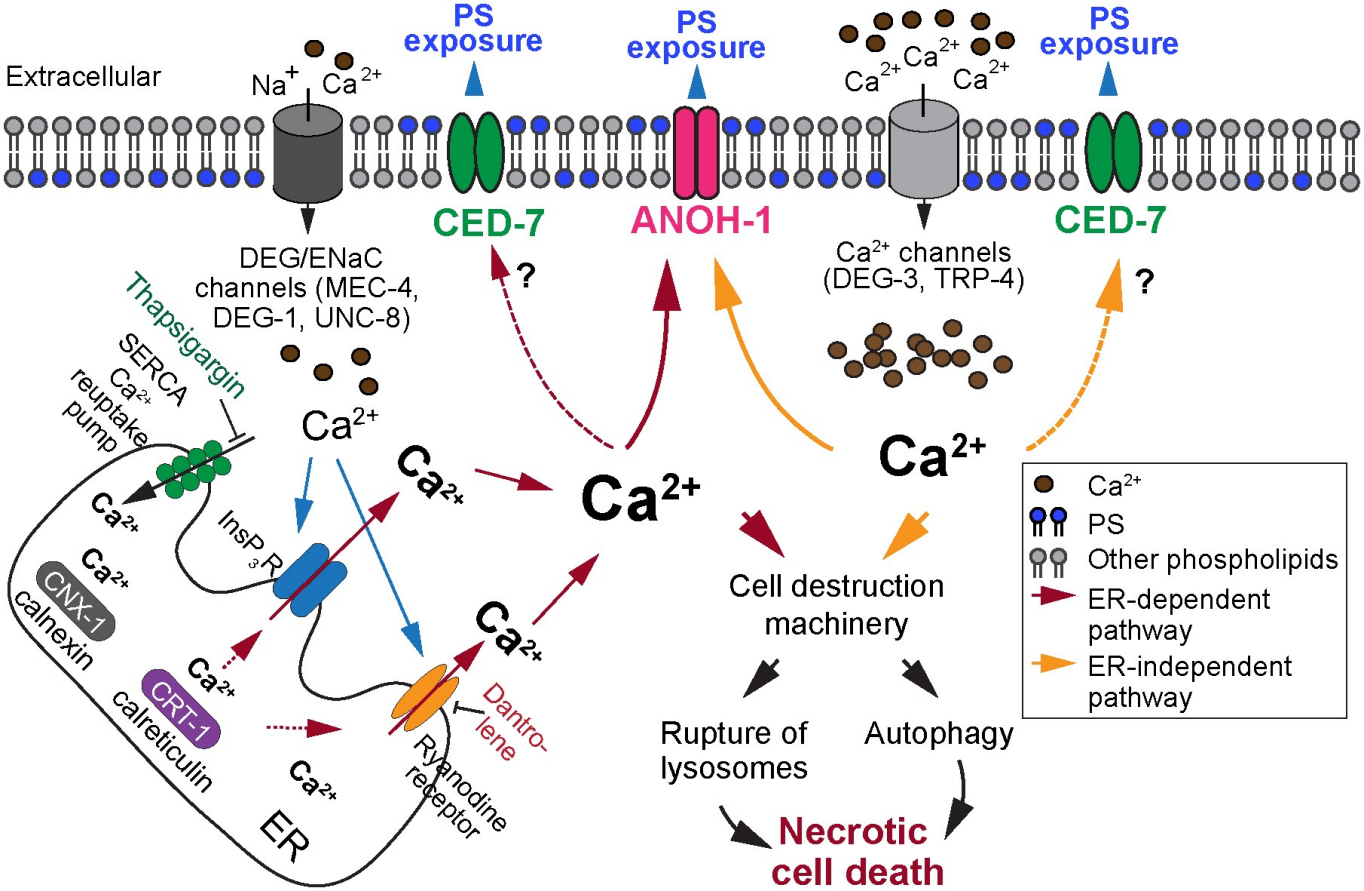
Two models illustrating how the increase of cytoplamic Ca^2+^ induces the exposure of PS on the surface of necrotic cells. See Discussion for a more detailed explanation of the models. The cylinders embedded in the plasma membrane represent various types of ion channels. Blue arrows indicate the Ca^2+^-induced Ca^2+^ release from the ER. Burgundy arrows indicate the ER-dependent Ca^2+^ accumulation pathway. Yellow arrows indicate the ER-independent Ca^2+^ increase pathway. The establishment of the calcium pool in the ER requires the calcium chaperons calreticulin (CRT-1) and calnexin (CNX-1). The release of Ca^2+^ from the ER requires the ryanodine receptor, whose activity is inhibited by dantrolene, and the InsP_3_ receptor (InsP_3_R). SERCA is an ER-surface Ca^2+^ reuptake pump whose function is inhibited by thapsigargin. Dominant mutations of DEC/ENaC channel subunits allow a small amount of Ca^2+^ to enter neurons. This, in turn, triggers a “Ca^2+^-induced” Ca^2+^ release from the ER, results in a further increase of cytoplasmic Ca^2+^ concentration. The resulting surge in the level of the cytoplasmic Ca^2+^ activates multiple downstream targets to induce parallel necrosis events. Dominant mutations in Ca^2+^ channels DEG-3 or TRP-4 induce PS exposure independent of the ER Ca^2+^ pool, presumably due to the large amount of Ca^2+^ let in the cells by these constitutively open Ca^2+^ channels. ANOH-1 function is necessary for the efficient PS exposure triggered by both ER-dependent and ER-independent Ca^2+^ increase mechanisms and we propose ANOH-1 is a prime candidate for a Ca^2+^-target that facilitates PS exposure. In addition, question marks indicate that the increased cytoplasmic Ca^2+^ might also activate CED-7 or other unknown PS-externalization enzymes. Besides Ca^2+^, there might be other upstream signaling molecules that induce PS exposure.

Previously, we discovered that necrotic touch neurons in *C*. *elegans* larvae remain intact and actively expose PS on their outer surfaces in order to attract engulfing cells [19]. Our continuing investigation reported here reveals a previously unknown signaling mechanism that triggers the exposure of PS on the surface of necrotic cells. Despite its essential roles in many cellular events, Ca^2+^ was not known to act as a trigger for the clearance of necrotic cells. We developed a time-lapse recording approach and monitored the dynamic change of the cytoplasmic Ca^2+^ concentration both prior to and throughout the necrosis of touch neurons in developing *C*. *elegans* embryos. The Ca^2+^ signal in touch neurons has previously been measured in adults [65] but has not been monitored in real time or during embryonic development. Our newly-developed real time-recording protocol has revealed a rapid and transient increase of the cytoplasmic Ca^2+^ in the touch neurons prior to cell swelling, a feature of necrotic cells, and the subsequent presentation of PS on necrotic cell surfaces. Further quantitative analysis demonstrated a close correlation between the levels of cytoplasmic Ca^2+^ and PS exposure. We further discovered that the ER Ca^2+^ pool was necessary for the the robust increase in cytoplasmic Ca^2+^ and the consequential PS exposure during the excitotoxic necrosis of neurons induced by mutations of DEG/ENaC family of sodium channels. On the other hand, the ER contribution of Ca^2+^ appears to be dispensable for PS exposure induced by constitutively active mutations of two different Ca^2+^ channels. Based on these findings and on the known mechanisms that regulate the intracellular Ca^2+^, we propose a model describing two different Ca^2+^-triggered PS exposure-mechanisms occuring in neurons undergoing necrosis, one ER-dependent, the other ER-independent. Our results further suggest that both mechanisms target ANOH-1, a putative Ca^2+^-dependent phospholipid scramblase, for PS externalization (Fig 11).

### A Ca^2+^-triggered, ER-assisted, “two-step” mechanism that promotes the exposure of PS on the surface of necrotic neurons

The DEG/ENaC sodium channel in touch neurons, of which MEC-4 is a subunit, plays an essential role in the mechanosensation [13]. Biochemical studies have revealed that the MEC-4(d) mutant protein alters the property of this sodium channel, making it permeable to Ca^2+^ [36]. This altered permeability was detected when overexpressed in Xenopus oocytes or in *C*. *elegans* touch neurons, although only a modest permeability of Ca^2+^ was detected [36]. In *mec-4(d)* mutants, multiple lines of evidence indicate that a further increase of the level of cytoplasmic Ca^2+^, provided by the ER through the calcium-induced Ca^2+^ release mechanism, triggers necrosis [35,38].

The exposure of PS is a common feature observed on multiple types of necrotic neurons, including necrosis induced by hyperactive mutations in three DEG/ENaC channel subunits (MEC-4, DEG-1 and UNC-8) and two Ca^2+^ channels (DEG-3 and TRP-4) (Fig 1 and [19]). Our time-lapse observation of Ca^2+^ signal intensity in *mec-4(d)* mutants has provided direct evidence of a transient increase in the cytoplasmic Ca^2+^ level prior to necrotic cell swelling and PS exposure. We further provide multiple lines of evidence to indicate that, similar to cell swelling, the exposure of PS on the surface of necrotic neurons in *mec-4(d)*, *deg-1(d)*, *and unc-8(d)* mutants is also regulated by the ER Ca^2+^ release. First, dantrolene, which inhibits the release of Ca^2+^ from the ER, attenuates PS exposure. Secondly, the *crt-1* mutations that block the establishment of ER Ca^2+^ pool result in the great reduction of PS exposure. Calreticulin (CRT-1) is an ER-localized calcium chaperon that plays a critical role in establishing the Ca^2+^ pool in the ER by binding to Ca^2+^ with high capacity [52]. Upon upstream signals, such as the binding of the InsP_3_ receptor (InsP_3_R) by cytoplasmic Ca^2+^, more Ca^2+^ is released from ER chaperons such as calreticulin and calnexin and enter the cytoplasm [37]. By monitoring the Ca^2+^ signal intensity over time in *crt-1(null); mec-4(d)* double mutant embryos, we observed very low cytoplasmic Ca^2+^ intensity in the PLM neurons. This evidence verified the lack of Ca^2+^ release from the ER despite the presence of the necrosis signal, presumably due to the lack of a normal Ca^2+^ pool in the ER. The *crt-1* null mutation severely reduced PS exposure in *deg-1* and *unc-8* mutants, indicating the essential role of a high level of cytoplasmic Ca^2+^ in inducing PS exposure. Thirdly, on the contrary, the thapsigargin treatment that inhibits reuptake of Ca^2+^ into ER not only induces necrosis of cells in otherwise wild-type animals, but also triggers the exposure of PS on the surfaces of these necrotic cells. Together, these results lead us to propose a “two-step” model for extracellular Ca^2+^ to induce PS exposure in response to hyperactive mutations of DEG/ENaC sodium channel subunits: step 1, a modest level of Ca^2+^ leaks into neurons through the mutated DEG/ENaC sodium channels, and step 2, these Ca^2+^ molecules activate the “Ca^2+^-triggered Ca^2+^ release” from the ER, which further increases the cytoplasmic Ca^2+^ level; once the cytoplasmic Ca^2+^ level reaches a threshold, necrosis and PS exposure are induced (Fig 11).

Is the exposure of PS merely a consequence of necrosis? Our further experiments demonstrate that this is not the case.

First of all, while low doses of dantrolene reduce the intensity of PS on the necrotic cell surfaces without suppressing cell swelling, higher doses both partially suppress cell swelling and further reduce PS intensity on the surfaces of the remaining necrotic cells (Fig 3). This phenomenon suggests that the threshold of cytoplasmic Ca^2+^ required for PS exposure is higher than that needed for inducing cell swelling, suggesting that PS exposure and cell swelling might be regulated by different calcium effectors. Secondly, we directly examined whether PS exposure is a downstream consequence of necrosis by blocking *mec-4(d)*-induced necrosis, through either inhibiting autophagy or impairing proper lysosomal function. As the execution of necrosis requires both lysosomes and the autophagy pathway [58–60], inhibiting autophagy or impairing proper lysosomal function each only suppresses cell swelling in a certain percentage of touch neurons. We observed that even when cell swelling was blocked, PS was still frequently detected on the plasma membrane of these normal-looking neurons. These observations indicate that blocking cell swelling does not necessarily block PS exposure, and further strongly suggest that the cytoplasmic Ca^2+^ targets multiple effectors in parallel, one of which leads to PS exposure. Similarly, thapsigargin-treated platelets expose PS on their surfaces but do not undergo cell death, supporting the notion that PS exposure is not dependent on cell death [56].

### We have also discovered a Ca^2+^-dependent, ER-independent mechanism that triggers PS exposure

It would stand to reason that if the Ca^2+^ influx from the extracellular space is in a large enough amount, the ER-dependent Ca^2+^-release could be dispensible for PS exposure. Among the insults that are known to trigger the necrosis of neurons, a dominant mutation in *deg-3*, which encodes a subunit of a ligand-gated calcium channel belonging to the nicotinic acetylcholine receptor family [43], induces neuronal necrosis in an ER-independent manner [35]. The *deg-3(u662)* dominant mutation results in a calcium channel with increased conductivity [43,66]. In addition to *deg-3*, *trp-4* encodes a protein belonging to the TRPN subfamily of TRP channels, which are Ca^2+^ channels [62]. We found that blocking the establishment of an ER Ca^2+^ pool by a *crt-1* null mutation did not affect the exposure of PS on necrotic cells resulting from the *deg-3* or *trp-4* mutations, in contrast to that observed in the *deg-1* and *unc-8* mutant backgrounds. These results indicate that when there is a large enough amount of Ca^2+^ entering into the neurons through the hyperactive Ca^2+^ channels, the Ca^2+^-triggered PS exposure might be independent of the ER Ca^2+^ release (Fig 11).

### The putative phospholipid scramblase ANOH-1 is likely a direct target of the cytoplasmic Ca^2+^

What protein(s) externalizes PS in response to the increased levels of cytoplasmic Ca^2+^? One potential candidate is *C*. *elegans* ANOH-1. TMEM16F, the mammalian homolog of ANOH-1, is both a Ca^2+^-activated cation channel and a Ca^2+^-dependent phospholipid scramblase [24,25]. Despite that its activity has not associated with necrotic cells in mammals, TMEM16F is essential for the PS exposure on platelets in response to Ca^2+^ signaling without compromising plasma membrane integrity [24,26]. Although its biochemical activity has not been determined, *C*. *elegans* ANOH-1 might act as a phospholipid scramblase whose activity is dependent on its association with Ca^2+^, as the Ca^2+^-binding motif identified in TMEM16F is conserved in ANOH-1 [19,67–70]. We found that, like in *mec-4(d)* mutants, in worms treated with thapsigargin, a null mutation of *anoh-1* severely reduced the intensity of PS on the surfaces of necrotic cells. This observation provides strong evidence to suggest that the high level of cytoplasmic Ca^2+^ activates ANOH-1’s phospholipid scramblase activity. This activation might be through direct interaction between Ca^2+^ and ANOH-1 (Fig 11). Similarly, in *trp-4(d); crt-1(null)* mutants, PS exposure is also substantially reduced comparing to *trp-4(d)* single mutants, indicating that both of the two mechanisms that increase cytoplamsic Ca^2+^ levels target ANOH-1 to promote PS exposure (Fig 11).

Conversely, CED-7, the other protein important for PS exposure, is not known to bind Ca^2+^, neither is CED-7’s mammalian homolog ABCA1. It remains to be investigated whether CED-7 is also regulated by Ca^2+^ or by a different upstream signaling molecule (Fig 11). Given that CED-7 is important for PS exposure on the surface of both apoptotic and necrotic cells [19,20], it is possible that CED-7 can be activated by multiple upstream signals.

As to how Ca^2+^ mediates cell swelling, the Ca^2+^-activated protease calpain is a known target of necrosis signals, whose stimulated activity is associated with lysosomal rupture and the activation of autophagy, two major events that lead to many cell morphological changes and the degradation of cellular proteins during necrosis [71–74]. We propose that during the initiation of necrosis, the elevated cytoplasmic Ca^2+^ concentration activates, in parallel, a number of downstream targets that include the PS-exposure enzymes, calpain, and possibly other proteases, and in this manner induces cellular changes of multiple aspects, including PS exposure and cell swelling (Fig 11).

### Does intracellular Ca^2+^ facilitate PS exposure on the surface of apoptotic cells?

Previously, extracellular Ca^2+^ was reported to stimulate the exposure of PS on the surface of a T lymphocyte hybridoma cell line undergoing apoptosis [75]. A Ca^2+^-dependent PS exposure mechanism was suggested to be a general mechanism utilized by apoptotic cells of different identities [76]; however, lines of evidence that do not support this hypothesis also exist [77,78]. Nonethless, it was unknown whether Ca^2+^ is involved in the developmentally programmed apoptosis and/or the subsequent exposure of the “eat me” signal in *C*. *elegans*. We determined that neither the number of apoptosis events nor the level of PS exposure is compromised during embryogenesis in *crt-1* mutants. These results indicate that the fluctuation of the cytoplasmic Ca^2+^ level does not trigger either apoptosis or PS exposure in developing *C*. *elegans* embryos. This is consistent with our previous finding that the proposed Ca^2+^-dependent phospholipid scramblase ANOH-1 is not involved in the PS exposure on apoptotic cells [19]. Together, our findings demonstrate that apoptotic and necrotic cells regulate the exposure of “eat me” signals and their subsequent clearance through distinct mechanisms; apoptotic cells utilize Ca^2+^-independent mechanisms, while necrotic cells utilize Ca^2+^-dependent mechanisms.

### The Ca^2+^-dependent regulatory mechanism for PS exposure is likely conserved throughout evolution

The regulatory mechanisms we have discovered in *C*. *elegans* might be mechanisms that are also employed in other organisms including mammals, particularly since the mammalian Ca^2+^-dependent scramblase TMEM16F is a close homolog of *C*. *elegans* ANOH-1. Whether TMEM16F plays a role similar to *C*. *elegans* ANOH-1 in facilitating the clearance of necrotic cells remains to be tested, although it is clear that TMEM16F is not involved in the PS exposure on apoptotic cells [79]. Insults that increase the cytoplasmic Ca^2+^ trigger the necrosis of neurons, glial cells, cardiomyocytes, cancer cells, and other cells [6,34,80–82], and it is possible that some or all types of these necrotic cells all utilize the Ca^2+^-dependent mechanism to activate the PS-exposure activity. We predict that the Ca^2+^-triggered PS exposure might be an evolutionarily conserved mechanism that facilitates the clearance of different types of necrotic cells. As calcium overload and the consequential excitotoxic necrosis play critical roles in the pathology of many diseases including neurodegenerative disorders, stroke, heart failure, and cancer [72,80–82], investigating the clearance of necrotic cells using *C*. *elegans* as a model organism will shed light on the pathology and therapeutics of these human diseases. For example, there are pre-clinical therapeutic attempts to use chemical inhibitors of the Ca^2+^-dependent protease calpain to hamper the necrosis occurring during pathological conditions such as brain ischemia [83]. What we have found suggests that this approach might not be able to efficiently inhibit the exposure of PS on cells whose necrosis is blocked and thus might not necessarily prevent these cells from being engulfed.

## Materials and methods

### Mutations, plasmids, strains, and transgenic arrays

*C*. *elegans* was grown at 20°C as previously described [84] unless indicated otherwise. The N2 Bristol strain was used as the wild-type strain. Mutations are described in [85] and in the Wormbase (http://www.wormbase.org) unless noted otherwise: LGI, *ced-1(e1735)*, *trp-4(ot337)*. LGII, *enIs74[*P_*dyn-1*_*mfg-e8*::*mCherry*, P_*mec-7*_ *gfp*, and *punc-76(+)]* (this study). LGIII, *anoh-1(tm4762)*, *crt-1* (*bz29 and bz50*). LGIV, *unc-8(n491sd); enIs77[*P_*ced-1*_*mfg-e8*::*mKate2* and *punc-76(+)]* (this study). LGV, *unc-76(e911)*, *deg-3(u662)*, *unc-51(e369)*, *enIs92[*P_*mec-7*_*GCaMP5G*, P_*dyn-1*_*mfg-e8*::*mCherry*, and *punc-76(+)]* (this study); LGX, *deg-1(u38ts)*, *mec-4(e1611)*.

Plasmids P_*dyn-1*_*mfg-e8*::*mCherry* and P_*mec-7*_*gfp* (pPD117.01, a gift from Andrew Fire) have been reported in [19]. P_*ced-1*_*mfg-e8*::*mKate2* was generated by first replacing the *dyn-1* promoter (P_*dyn-1*_) [86] in P_*dyn-1*_*mfg-e8*::*mCherry* with P_*ced-1*_ [23] and then replacing mCherry cDNA in P_*ced-1*_*mfg-e8*::mCherry with mKate2 cDNA [87]. P_mec*-7*_*GCaMP5G* was generated by cloning the GCaMP5G cassette obtained from pCMV-GCaMP5G [47] to the BamH1 and EcoR1 sites of pPD117.01. Plasmid P_*crt-1*_*NLS-gfp* was generated by amplifying the genomic DNA that covers the first 6 amino acids including the start codon and a 1.5kb upstream region representing the *crt-1* promoter (P_*crt-1*_) [88] and cloning this fragment into the Sph1 and BamH1 sites of pPD95.69 (a gift from Andrew Fire), in frame with the nuclear localization signal (NLS) tagged GFP coding sequence, generating a NLS-GFP reporter expressed under the control of P_*crt-1*_. Plasmid P_*crt-1*_*NLS-mNeonGreen* was constructed by replacing the coding sequence for GFP with that for mNeonGreen [89].

Extrachromosomal arrays were generated by microinjection [90] of plasmids with co-injection marker p*unc-76(*+*)* [91] into strains carrying the *unc-76(e911)* mutation. Transgenic animals were isolated as non-Unc animals. *enIs74*, *enIs77*, and *enIs92* are integrated transgenic arrays [90] generated from the corresponding transgenic arrays in this study. *trp-4(ot337)*, *crt-1*(*bz29)*, *crt-1(bz50)*, *unc-8(n491sd)*, and *deg-1(u38)* were provided by the *C*. *elegans* Genetic Center (CGC).

Standard genetic crosses [84] were used for generating double and triple mutant strains except for the following three double mutants that carry *enIs74*: (1) *unc-8(n491sd); crt-1(bz29)*; (2) *deg-3(u662) crt-1(bz29)*; and (3) *trp-4(ot337)*; *crt-1(bz29)*. These three strains were generated by CRISPR/Cas9-mediated gene editing of the *bz29* mutation into the corresponding *unc-8*, *deg-3*, and *trp-4* single mutants carrying *enIs74*. The *crt-1(bz29)* mutation is a change of nucleotide 83 (the 1^st^ nucleotide of the start codon is designated as nucleotide 1) from G to A, resulting in a Trp28-to-stop codon nonsense mutation [35]. We used the Paix protocol [92] to introduce the G83A mutation into each of the three above single mutant strain. The tracer RNA and crRNAs, and the Cas9 protein, were from Integrated DNA technologies (IDT), Inc. The repair template oligo and other oligos were ordered from Sigma-Aldrich, Inc. Sequencing results confirmed the expected changes as homozygous in the three corresponding double mutant strains.

### Chemical treatments of *C*. *elegans*

#### Dantrolene and thapsigargin treatments

Worms were treated with various doses of dantrolene (Tocris Bioscience, Inc.) as described previously [35]. Dantrolene was dissolved in DMSO and was both spread on unseeded NGM plates and added to the suspension of OP50 (the *E*. *coli* strain seeded onto the NGM plates). One day after seeding the NGM plates with the drugged OP50, L4 hermaphrodites were placed onto the dantrolene plates and raised at room temperature (20–22°C). Twenty-four to 36 hrs later, L1 larvae (F1 progeny) hatched within 1 hr were examined by both DIC and fluorescence imaging. Dantrolene is likely affecting embryos through entry into the germline of adult hermaphrodites and subsequently being incorporated in the embryos. The 0 μM dantrolene control sample represent worms from NGM plates and seeded OP50 that were treated with DMSO, the solvant for dantrolene. The thapsigargin (Sigma-Aldrich, Inc.) treatment protocol is similar to that of dantrolene treatment, except that the worms were raised at 25°C instead of room temperature (20–22°C).

#### NH_4_Cl treatment

As described previously [35], 1M NH_4_Cl (Sigma-Aldrich, Inc.) solution in water was added to 10 mL S medium containing 50 young adult hermaphrodites and appropriate amount of concentrated bacteria OP50 (food for worms) to reach a final concentration of 5 mM. This suspension was grown at room temperature and with constant shaking. Fifteen hours later, young L1 larvae were collected and analyzed by DIC and fluorescence microscopy. Note that because the progeny of the young adults were grown in the liquid culture and could not be distinctly timed, the L1 larvae that we collected and analyzed are between 1–3 hrs after hatching. As a consequence, the mean number of necrotic PLMs observed in the tail is slightly lower than that of the L1 larvae scored within 1 hr of hatching due to the extra time after hatching.

### DIC microscopy and scoring the number of necrotic cells and apoptotic cells

DIC microscopy was performed using an Axioplan 2 compound microscope (Carl Zeiss) equipped with Nomarski DIC imaging apparatus, a digital camera (AxioCam MRm; Carl Zeiss), and imaging software (AxioVision; Carl Zeiss), or with an Olympus IX70-Applied precision DeltaVision microscope equipped with a DIC imaging apparatus, a Photometrics Coolsnap 2 digital camera, and the SoftWoRx imaging software (GE Healthcare, Inc.). In *ced-1(e1735); mec-4(e1611)* mutant and *ced-1(e1735); mec-4(+)* control backgrounds, the presence of two necrotic PLM neurons (PLML and PLMR) in the tail was scored by their swelling morphology in L1 larvae as previously described [93]; these larvae were collected within 1 hr after hatching, or within 1-3hrs after hatching, depending on what is annotated in the figure legends. Between 30–60 L1 larvae were scored for each sample. These larvae were grouped as 10 animals per group. The mean values of the number of necrotic PLM neurons of each group were calculated, and the mean values and standard errors of the means of all groups were presented in bar graphs. To examine *deg-1(u38ts)*-induced necrosis, adults were grown at 25°C for one day, at which point we collected the embryos that they laid. These embryos were incubated at 25°C and allowed to hatch, and we examined the L1 larvae within 1hr after they hatched. Between 50–129 larvae were scored for the number of necrotic cells in the head region. These larvae were grouped as 10, 15, and 25 animals per group for the *deg-1(u38ts)*, *deg-1(u38ts); crt-1(bz50)*, and *deg-1(u38ts); crt-1(bz29)* strains, respectively. The mean values of the number of necrotic cells of each group were calculated, and the mean values and standard errors of the means of all groups were presented in bar graphs.

The number of apoptotic cells was scored in the head of newly hatched L1 larvae as described in [93].

The numerical values of the graphs can be found in S1 Data.

### Fluorescence microscopy, time-lapse recording, and quantification of image intensity

An Olympus IX70-Applied Precision DeltaVision microscope equipped with a DIC imaging apparatus and a Photometrics Coolsnap 2 digital camera was used to acquire serial Z -stacks of fluorescence and DIC images, whereas the SoftWoRx software was utilized for image deconvolution and processing as described in [19,93]. To quantify the intensity of PS signal on the surface of a necrotic PLM neuron, the cell surface area containing the MFG-E8::mCherry signal was outlined by two closed polygons. The total signal inetensity as well as the area of each polygon were recorded. The unit PS signal intensity (PS_PLM_) of the “donut shape” area between the two polygons was calculated as follows: UPS_PLM_ = (PS_externalpolygon_−PS_internalpolygon_)/(Area_externalpolygon_−Area_internalpolygon_). The background mCherry signal (PS_Background_) of a “circlular” area nearby and the area size (Area_background_) were obtained and the unit intensity UPS_background_ = PS_background_/Area_background_. The relative PS signal intensity (RPS_PLM_) = UPS_PLM_ / UPS_background_. According to this formular, the value 1 indicates no enrichment of signal comparing to the non-specific background signal. The PS intensity on the surfaces of other necrotic neurons, living PLM neurons, and apoptotic cells were also measured using the above method.

To quantify the Ca^2+^ intensity in the cytoplasm of live or necrotic PLM neurons, the cell body of a PLM neuron was outlined by a polygon and the GCaMP5G signal value within the polygon was obtained and referred to as Ca_x_. Ca_Background_ of an area in equivalent size in a neighboring region was similarly obtained. The relative Ca^2+^ intensity of a particular cell was defined as Ca_R_ = Ca_x_ / Ca_Background_.

To monitor the dynamics of Ca^2+^ release into the cytoplasm of PLM neurons, cell swelling during necrosis, and PS presentation during embryogenesis, we modified a previously established protocol [19]. We mounted embryos on an agar pad on a glass slide with M9 buffer. The recording period covered 560 min (100 min after worms reached the 2-fold stage (460min)) to 900 min post-1^st^ embryonic cleavage. Serial Z stacks were captured in 10–15 sections at 0.5μm per section. Recording interval was 2.5 min from 560–680 min post-1^st^ embryonic cleavage and 5 min from 685–900 min post 1^st^ cleavage. Unfortunately, since embryos being recording are constantly moving inside the eggshell, images recorded in most of the time points do not have the PLML or PLMR neurons in focus. Thus images useful for signal quantification are far fewer than those captured. We set the minimum time points required for constructing a curve to 10 per embryo. Each curve used in our integral analysis includes between 10 and 20 time points. The numerical values of the graphs and plots can be found in S1 Data.

## Supporting information

S1 Fig Additional examples of time-lapse plots of the dynamic Ca^2+^ and PS signal levels throughout mid- to late-embryogenesis. *(Related to Fig 2)* (A-C) Reproted here are results of three time-lapse series recording cytoplasmic Ca^2+^ levels and PS signal in or on the surface of PLML and PLMR neurons, respectively. Embryos are *mec-4(e1611)* homozygotes carrying the *enIs92* transgenic array. Relative signal levels (in comparison to the background levels) of GCaMP5G in the cytoplasm, and of MFG-E8::GFP on the membrane surface, of the PLM neurons are ploted over time. The green, blue, and red arrows underneath the X-axis mark the time points when the rise of Ca^2+^ signal, the distinct cell swelling morphology becomes obvious, and PS is first seen on the surface of the neuron, respectively, by eye observation. (D) The mean integral values of relative cytoplasmic Ca^2+^ intensities in each cell measured from 4 necrotic PLM neurons in *mec-4(e1611)* embryos and 4 live PLM neurons in *mec-4(+)* embryos were obtained by calculating the integral value of GCaMP5G intensities within three time periods, 560–800 min, the entire time-lapse recording period, 560–695 min, the period prior to the mean time point when cell swelling is obvious, and 696–800 min post-1^st^ embryonic division, respectively. Error bars represent s.e.m. (E) Time-lapse DIC images following the necrosis of one PLML neuron (white arrows). Yellow arrows mark the PLMR neuron which went out of focus in later time points. Time points are marked as min post-first embryonic cell division. Recording started at 560 min and ended at 900 min. The PLML neuron starts swelling at 675 min and continues swelling in the later time points until reaching its maximal size at 870 min. The scale bar is 10μm.
(TIF)

S2 Fig The relative Ca^2+^ signal intensity over time in live or necrotic PLM neurons in different genotypes and/or under dantrolene treatment. *(Related to Figs 2 and 4)* Presented here are results of time-lapse recording experiments that measure the intensity of cytoplasmic Ca^2+^ and cell swelling of PLM neurons during embryonic development. All embryos carry the transgenic array expressing P_*mec-7*_*GCaMP5G*. (A) DIC and fluorescence time-lapse images of two live PLM neurons in one *mec-4(+)* embryo. Time points are marked as min post-1^st^ embryonic division. White and yellow arrowheads mark the PLML and PLMR neurons, respectively. Scale bars are 10μM. (B) The relative signal levels of GCaMP5G were measured in 4 live PLM neurons and plotted over time. Graph (a) displays the plots of the PLML and PLMR neurons shown in (A), whereas graph (b) displays the plots of the PLML and PLMR neurons in an additional embryo. (C) The relative signal levels of GCaMP5G were measured in two necrotic PLM neurons in a *mec-4(e1611)* mutant embryo from a plate treated with DMSO but no dantrolene. Two other examples are shown in Fig 4. (D) The relative GCaMP5G signal levels measured in 2 necrotic PLM neurons in *mec-4(e1611)* mutant embryos from a 3μM dantrolene treated plate are plotted over time.
(TIF)

S3 Fig A null mutation in *crt-1* blocks the rise of cytoplasmic Ca^2+^ in touch neurons. (*Related to Fig 5*) (A-B) Presented here are results of time-lapse recording experiments that measure the intensity of cytoplasmic Ca^2+^ and cell swelling of 4 PLM neurons during the development of two *crt-1(bz29); mec-4(e1611)* mutant embryos carrying the transgenic array expressing P_*mec-7*_*GCaMP5G*. The relative GCaMP5 intensity in living PLM neurons are plotted over time. (C) The mean integral values of relative cytoplasmic Ca^2+^ intensities in each cell measured from 4 necrotic PLM neurons in *mec-4(e1611)* embryos and 4 PLM neurons whose necrosis were suppressed in *crt-1(bz29)*; *mec-4(e1611)* embryos were obtained by calculating the integral value of GCaMP5G intensities within three time periods, 560–800 min, the entire time-lapse recording period, 560–695 min, the period prior to the mean time point when cell swelling is obvious, and 696–800 min, the period after cell swelling, respectively. Error bars indicate s.e.m.
(TIF)

S4 Fig NH_4_Cl partially suppresses necrosis but allows the exposure of PS on live PLM neurons. *(Related to Fig 7)* (A) The NH_4_Cl treatment partially suppresses necrosis induced by the *mec-4(e1611)* mutation. The mean numbers of PLM neurons (labeled with P_*mec-7*_GFP) that display the swelling necrosis phenotype in the tails of young *ced-1(e1735); mec-4(e1611)* L1 larvae from liquid cultures treated or not treated with 5mM NH_4_Cl are presented in the graph. Bars represent the mean values of each sample. Error bars indicate s.e.m. The numbers in the parentheses represent the numbers of L1 larvae scored. “**”, 0.001<p<0.01, Student *t*-test. (B) Images of necrotic and live PLM neurons that present PS on their outer surfaces in *mec-4(e1611)* L1 larvae. L1 larvae were from liquid cultures either treated with 5mM NH_4_Cl (d-f, white arrowheads) or left untreated (a-c). PLM neurons (c (white arrow), f (white and yellow arrowheads)) are labeled with the P_*mec-7*_GFP reporter. DIC images identify one necrotic PLM neuron (white arrow) and one apoptotic cell (yellow arrow) in the tail of an L1 larva not treated with NH_4_Cl (a), and one necrotic PLM (yellow arrowhead) and one live PLM (white arrowhead) in an L1 larva from the 5mM NH_4_Cl treated culture (d-f). PS presentation on the cell surfaces (b, e) is detected by MFG-E8::mCherry. Scale bars are 5μm. (C) A bar graph representing the percentage of live PLM neurons that expose PS on their surfaces among all living PLM neurons in early L1 larvae after NH_4_Cl treatment. Error bars represent s.e.m.. The numbers in the parentheses represent the numbers of living cells scored.
(TIF)

S1 Data This Excel file includes all the quantitative data used for generating the curves and graphs in the figures. (XLSX)
