# Supplementary figures and images for: Calcium ions trigger the exposure of phosphatidylserine on the surface of necrotic cells
Source: PLoS Genet. 2021 Feb 11;17(2):e1009066. doi: 10.1371/journal.pgen.1009066 (PMC7904182; doi:10.1371/journal.pgen.1009066)

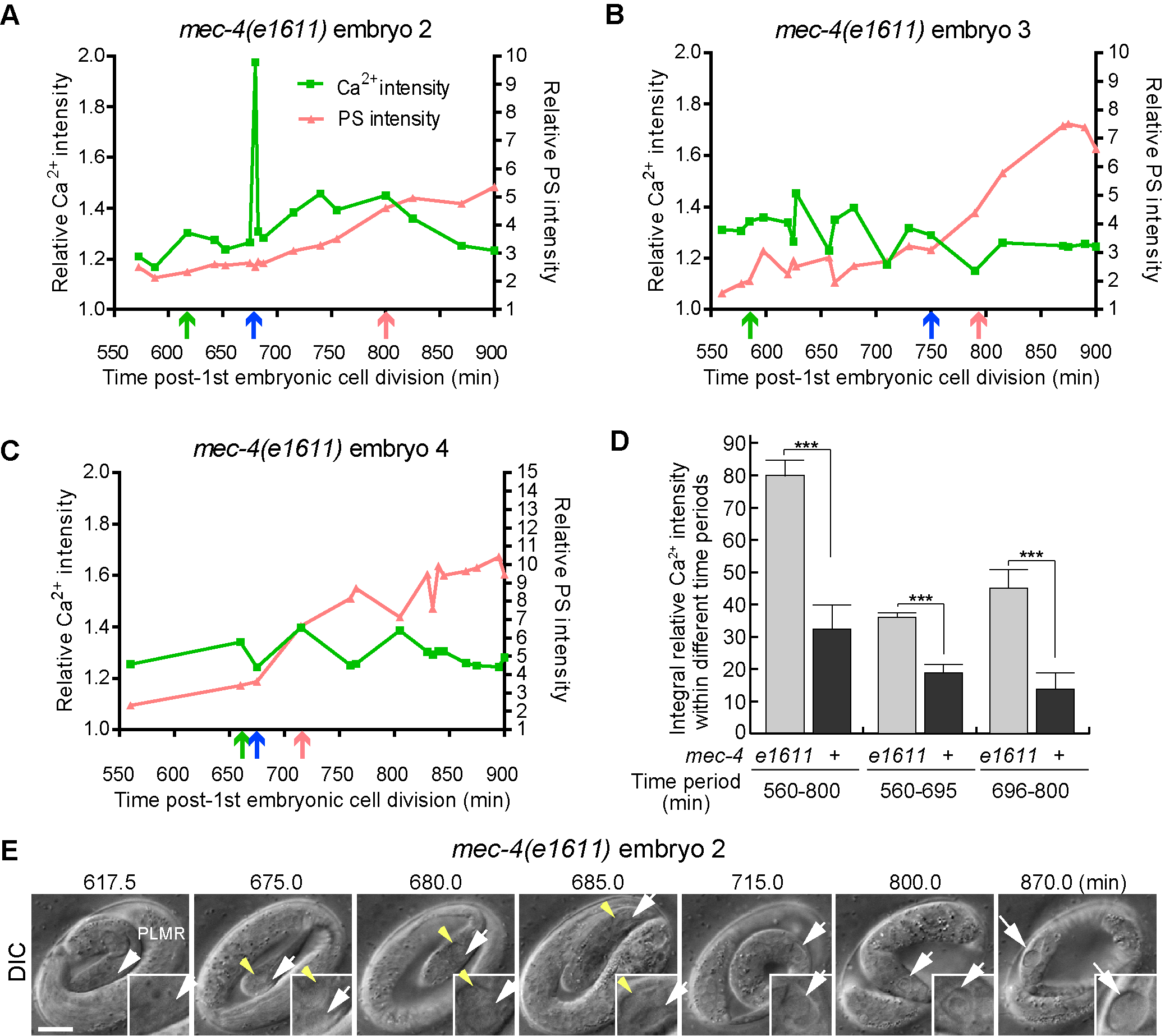

Supplement: S1 Fig — (Related to Fig 2) (A-C) Reproted here are results of three time-lapse series recording cytoplasmic Ca2+ levels and PS signal in or on the surface of PLML and PLMR neurons, respectively. Embryos are mec-4(e1611) homozygotes carrying the enIs92 transgenic array. Relative signal levels (in comparison to the background levels) of GCaMP5G in the cytoplasm, and of MFG-E8::GFP on the membrane surface, of the PLM neurons are ploted over time. The green, blue, and red arrows underneath the X-axis mark the time points when the rise of Ca2+ signal, the distinct cell swelling morphology becomes obvious, and PS is first seen on the surface of the neuron, respectively, by eye observation. (D) The mean integral values of relative cytoplasmic Ca2+ intensities in each cell measured from 4 necrotic PLM neurons in mec-4(e1611) embryos and 4 live PLM neurons in mec-4(+) embryos were obtained by calculating the integral value of GCaMP5G intensities within three time periods, 560–800 min, the entire time-lapse recording period, 560–695 min, the period prior to the mean time point when cell swelling is obvious, and 696–800 min post-1st embryonic division, respectively. Error bars represent s.e.m. (E) Time-lapse DIC images following the necrosis of one PLML neuron (white arrows). Yellow arrows mark the PLMR neuron which went out of focus in later time points. Time points are marked as min post-first embryonic cell division. Recording started at 560 min and ended at 900 min. The PLML neuron starts swelling at 675 min and continues swelling in the later time points until reaching its maximal size at 870 min. The scale bar is 10μm. (TIF) [file pgen.1009066.s001.tif]

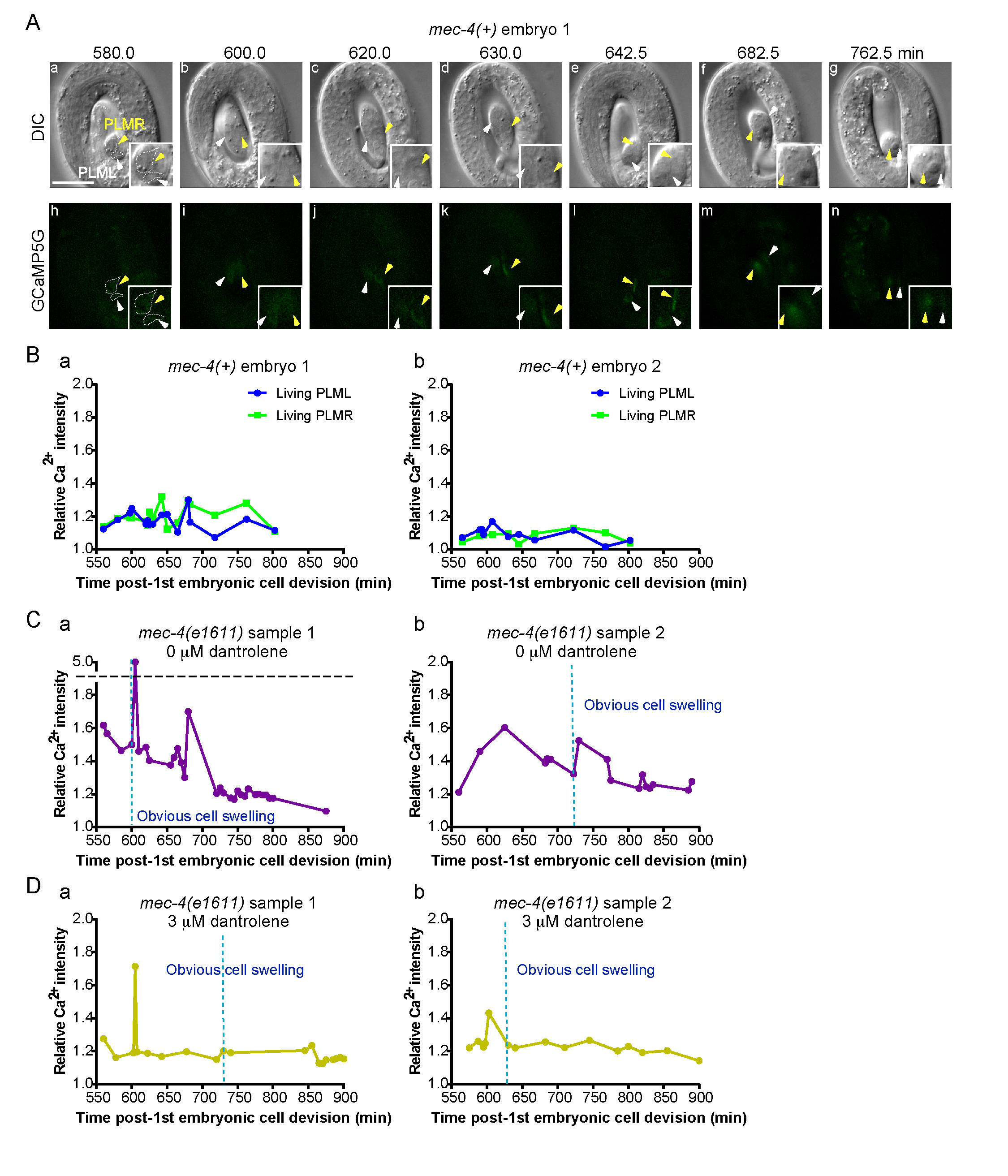

Supplement: S2 Fig — (Related to Figs 2 and 4) Presented here are results of time-lapse recording experiments that measure the intensity of cytoplasmic Ca2+ and cell swelling of PLM neurons during embryonic development. All embryos carry the transgenic array expressing Pmec-7GCaMP5G. (A) DIC and fluorescence time-lapse images of two live PLM neurons in one mec-4(+) embryo. Time points are marked as min post-1st embryonic division. White and yellow arrowheads mark the PLML and PLMR neurons, respectively. Scale bars are 10μM. (B) The relative signal levels of GCaMP5G were measured in 4 live PLM neurons and plotted over time. Graph (a) displays the plots of the PLML and PLMR neurons shown in (A), whereas graph (b) displays the plots of the PLML and PLMR neurons in an additional embryo. (C) The relative signal levels of GCaMP5G were measured in two necrotic PLM neurons in a mec-4(e1611) mutant embryo from a plate treated with DMSO but no dantrolene. Two other examples are shown in Fig 4. (D) The relative GCaMP5G signal levels measured in 2 necrotic PLM neurons in mec-4(e1611) mutant embryos from a 3μM dantrolene treated plate are plotted over time. (TIF) [file pgen.1009066.s002.tif]

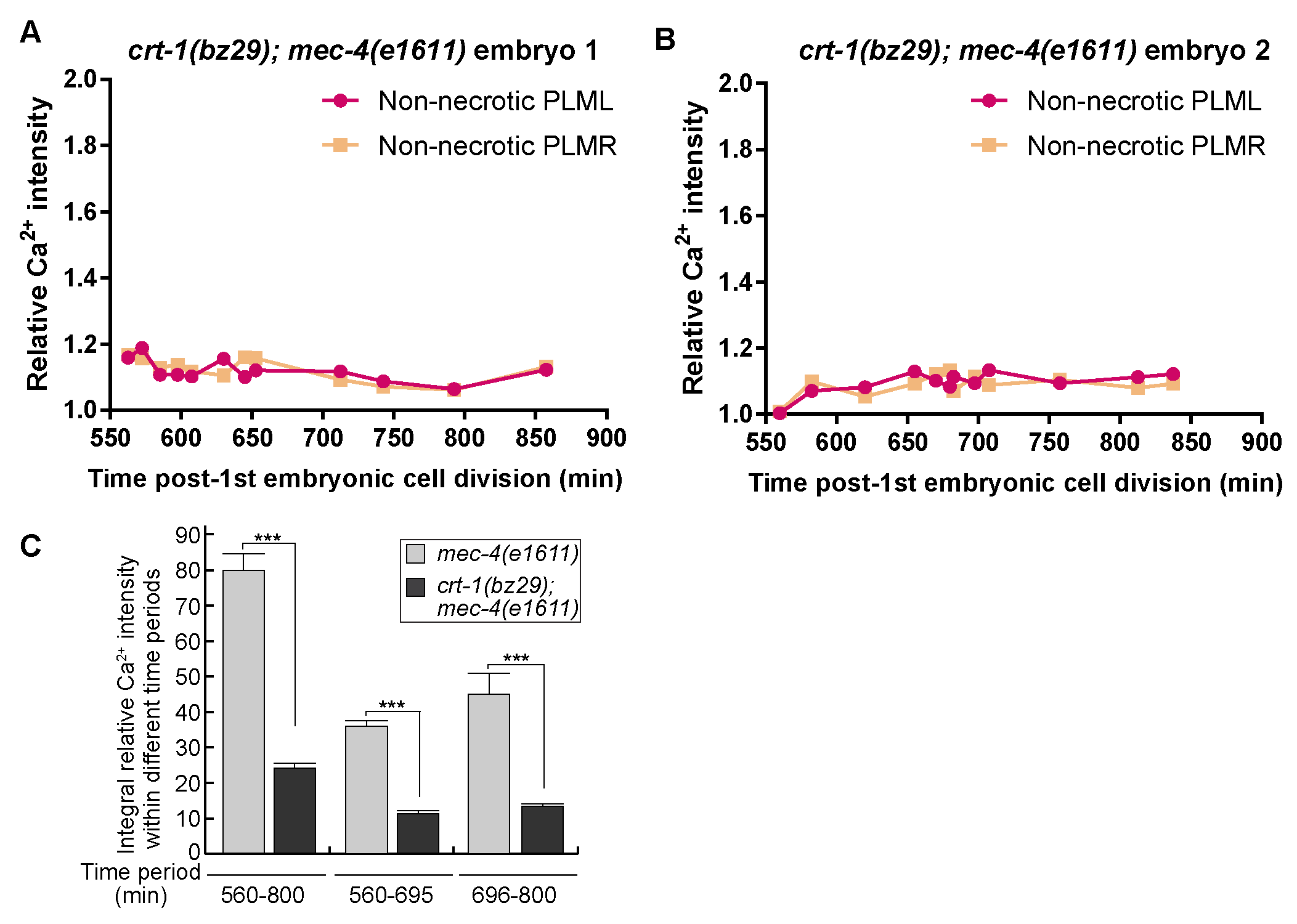

Supplement: S3 Fig — (Related to Fig 5) (A-B) Presented here are results of time-lapse recording experiments that measure the intensity of cytoplasmic Ca2+ and cell swelling of 4 PLM neurons during the development of two crt-1(bz29); mec-4(e1611) mutant embryos carrying the transgenic array expressing Pmec-7GCaMP5G. The relative GCaMP5 intensity in living PLM neurons are plotted over time. (C) The mean integral values of relative cytoplasmic Ca2+ intensities in each cell measured from 4 necrotic PLM neurons in mec-4(e1611) embryos and 4 PLM neurons whose necrosis were suppressed in crt-1(bz29); mec-4(e1611) embryos were obtained by calculating the integral value of GCaMP5G intensities within three time periods, 560–800 min, the entire time-lapse recording period, 560–695 min, the period prior to the mean time point when cell swelling is obvious, and 696–800 min, the period after cell swelling, respectively. Error bars indicate s.e.m. (TIF) [file pgen.1009066.s003.tif]

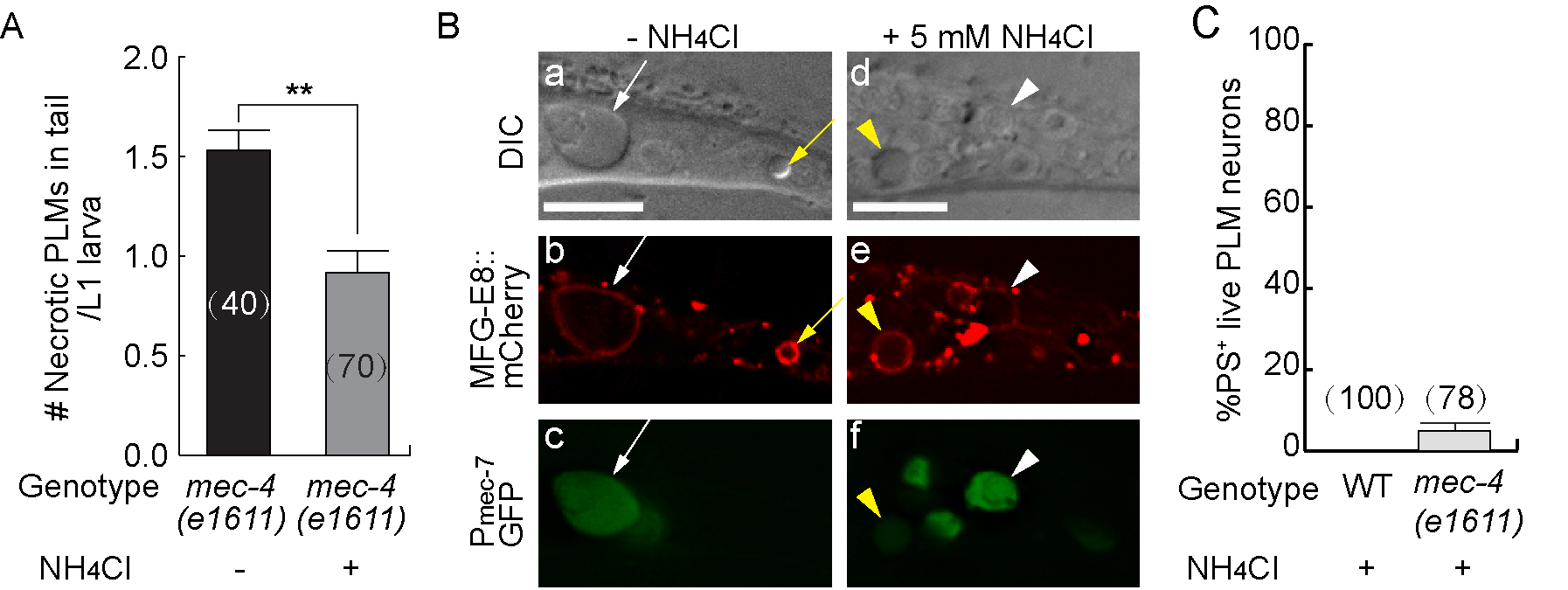

Supplement: S4 Fig — (Related to Fig 7) (A) The NH4Cl treatment partially suppresses necrosis induced by the mec-4(e1611) mutation. The mean numbers of PLM neurons (labeled with Pmec-7GFP) that display the swelling necrosis phenotype in the tails of young ced-1(e1735); mec-4(e1611) L1 larvae from liquid cultures treated or not treated with 5mM NH4Cl are presented in the graph. Bars represent the mean values of each sample. Error bars indicate s.e.m. The numbers in the parentheses represent the numbers of L1 larvae scored. “**”, 0.001<p<0.01, Student t-test. (B) Images of necrotic and live PLM neurons that present PS on their outer surfaces in mec-4(e1611) L1 larvae. L1 larvae were from liquid cultures either treated with 5mM NH4Cl (d-f, white arrowheads) or left untreated (a-c). PLM neurons (c (white arrow), f (white and yellow arrowheads)) are labeled with the Pmec-7GFP reporter. DIC images identify one necrotic PLM neuron (white arrow) and one apoptotic cell (yellow arrow) in the tail of an L1 larva not treated with NH4Cl (a), and one necrotic PLM (yellow arrowhead) and one live PLM (white arrowhead) in an L1 larva from the 5mM NH4Cl treated culture (d-f). PS presentation on the cell surfaces (b, e) is detected by MFG-E8::mCherry. Scale bars are 5μm. (C) A bar graph representing the percentage of live PLM neurons that expose PS on their surfaces among all living PLM neurons in early L1 larvae after NH4Cl treatment. Error bars represent s.e.m.. The numbers in the parentheses represent the numbers of living cells scored. (TIF) [file pgen.1009066.s004.tif]
